# Long-term vegetation dynamics in Spain’s National Park Network: insights from remote sensing data

**DOI:** 10.1007/s10661-025-14233-w

**Published:** 2025-06-19

**Authors:** Magí Franquesa, Maria Adell-Michavila, Sergio M. Vicente-Serrano

**Affiliations:** 1 Instituto Pirenaico de Ecología, Consejo Superior de Investigaciones Científicas (IPE-CSIC), 50059 Zaragoza, Spain; 2 Laboratorio de Climatología y Servicios Climáticos (LCSC), CSIC-Universidad de Zaragoza, Zaragoza, Spain

**Keywords:** Conservation management, Environmental monitoring, Spatiotemporal trends, Forest expansion, Landscape dynamics, Protected areas

## Abstract

Understanding long-term vegetation dynamics in protected areas is crucial for assessing ecosystem resilience and informing adaptive management strategies. This study presents a comprehensive analysis of vegetation trends across Spain’s National Park Network from 1984 to 2023, using Landsat imagery processed in Google Earth Engine. Twelve national parks, representing diverse biogeographical regions and ecosystems, were analyzed using vegetation indices such as NDVI, SAVI, kNDVI, and NDMI. The Mann–Kendall test and Theil-Sen slope estimator were employed to detect monotonic trends and quantify their magnitudes, respectively. Our results reveal a predominant increase in vegetation activity across most parks over the past four decades, though with notable spatial and seasonal variations influenced by topographic gradients, bioclimatic zones, and vegetation types. High-altitude parks exhibited strong seasonal dynamics, with positive trends concentrated during the growing season, whereas Mediterranean parks showed more consistent trends throughout the year. Conversely, wetland parks like Las Tablas de Daimiel displayed concerning negative trends, highlighting ecosystem vulnerabilities associated with hydrological stress. These findings underscore the importance of integrating high-resolution remote sensing data into long-term ecological monitoring programs to track ecosystem functioning and assess management practices in protected areas. Combining remote sensing with field observations is essential to support evidence-based conservation strategies in response to climate change and other anthropogenic pressures.

**Supplementary Information:**

The online version contains supplementary material available at 10.1007/s10661-025-14233-w.

## Introduction

Protected natural areas play a fundamental role in global biodiversity conservation by serving as refuges for threatened species, safeguarding critical ecosystem services, and mitigating the impacts of climate change (Bruner et al., 2001; Duncanson et al., 2023; Mi et al., 2023; Watson et al., 2014). Globally, networks of protected areas, such as national parks, significantly contribute to international conservation targets, including the Convention on Biological Diversity’s Aichi Targets and the United Nations Sustainable Development Goals (Secretariat of the Convention on Biological Diversity, 2020; United Nations, 2015). However, these areas face increasing pressures from climate change, land-use changes, and invasive species, which threaten their ability to maintain biodiversity and ecosystem resilience (Calvache et al., 2016; Gallardo & Capdevila-Argüelles, 2024; Kubacka et al., 2022; Mingarro & Lobo, 2023; Thomas et al., 2004; Zhao et al., 2024). Long-term monitoring of ecosystem dynamics within protected areas is therefore essential for informing adaptive management strategies and ensuring their effectiveness in a rapidly changing world.

Remote sensing has become an indispensable tool for monitoring protected areas, providing cost-effective, consistent, and large-scale data for assessing ecosystem functioning, land-use changes, and disturbances (Nagendra et al., 2013; N. Pettorelli et al., 2016; Nathalie Pettorelli, 2019; Y. Wang et al., 2020). It enables the monitoring of ecosystem processes, climate impacts, and conservation effectiveness through various metrics, such as vegetation indices and land-use classification (Rose et al., 2015). The growing application of satellite-based data in conservation research allows for comprehensive and repeatable assessments at multiple spatial and temporal scales, supporting biodiversity monitoring, climate impact assessments, land cover change detection, and evaluations of protected area effectiveness (Duan et al., 2020; Hernández-Romero et al., 2022).

Spain’s National Park Network, established in 1918 with the creation of Covadonga Mountain National Park (now known as Picos de Europa National Park) and Ordesa Valley National Park (currently named Ordesa y Monte Perdido National Park), encompasses some of the most biodiverse and geologically diverse landscapes in Europe (Salazar et al., 2014). From the wetlands and dune systems in Doñana to the alpine formations of Aigüestortes and Picos de Europa, these parks play a crucial role in preserving the country’s natural heritage. Remote sensing techniques have been widely applied to analyze various ecological aspects within these parks, including vegetation dynamics (Alcaraz-Segura, Baldi, et al. 2008a; Alcaraz-Segura, Cabello, et al., 2008a, 2008b; Alcaraz-Segura et al., 2009; Arrogante-Funes et al., 2024; Dionisio et al., 2012), forest cover changes (Ameztegui et al., 2021; Dinca et al., 2017), wetland hydrology (Díaz-Delgado et al., 2016), an land use-land cover (LULC) changes (Calvache et al., 2016; Hernández-Romero et al., 2022; Rodríguez-Rodríguez & Martínez-Vega, 2017). However, most of these studies rely on low-resolution sensors (e.g., MODIS, AVHRR), which lack the spatial detail required to effectively analyze Spain’s ecologically diverse national parks (Alcaraz-Segura, Baldi, et al. 2008b). Moreover, prior research has often focused on short time frames or a limited number of parks, leaving significant gaps in understanding long-term vegetation dynamics across the entire network.

Although low-spatial-resolution satellite data provide valuable insights into broad vegetation trends and offer high temporal frequency, they often lack the spatial detail needed to detect fine-scale heterogeneity within ecosystems, particularly in topographically complex and biologically diverse regions like the ecosystems of the Mediterranean region. The increasing availability of high-resolution datasets, such as 30-m Landsat imagery, helps overcome this limitation by enabling detailed monitoring of vegetation changes at local scales and capturing spatial variability driven by topography, bioclimatic gradients, and land uses. Platforms like Google Earth Engine (Gorelick et al., 2017) further enhance the capacity of ecosystem monitoring by enabling efficient processing of decades of satellite data and facilitating the generation of long-term time series of vegetation indices—such as NDVI, SAVI, kNDVI, and NDMI—which provide critical insights into vegetation productivity and moisture conditions, essential for understanding ecosystem dynamics (Bannari et al., 1995; Ceccato et al., 2002; Tucker, 1979).

Building on these advancements in remote sensing technology, ecosystem monitoring has been integrated into Spain’s National Park Network through initiatives such as the REMOTE system (Cabello-Piñar et al., 2016). This program tracks vegetation productivity, phenological trends, and anomalies across the park network, providing valuable insights through annual reports that inform conservation planning and decision-making (https://www.miteco.gob.es/es/parques-nacionales-oapn/red-parques-nacionales/seguimiento/seguimiento-ecologico/informes_productividad.html, last accessed January 2025). However, despite the contributions of current satellite-based monitor initiatives, primarily supporting park management, a significant gap remains in the scientific literature regarding detailed, long-term analyses of fine-scale vegetation dynamics.

This study addresses these gaps by providing a comprehensive assessment of vegetation trends across Spain’s national parks, offering scientific insights that complement existing monitoring efforts. Using Landsat data processed in Google Earth Engine, we leverage its 30-m spatial resolution to capture fine-scale variability in vegetation dynamics over the past 40 years. The analysis includes all national parks in mainland Spain and Balearic Islands, excluding those in the Canary Islands due to limitations in image availability.

By examining patterns of change across seasons, vegetation types, and topographic gradients—including slope orientation and bioclimatic characteristics— this research enhances our understanding of long-term vegetation trends. The findings provide critical insights into ecosystem vulnerabilities and support adaptive management strategies in response to climate change.

## Material and methods

### Study area

Spain’s National Park Network consists of 16 parks that represent the country’s diverse natural landscapes, ranging from the volcanic terrain of the Canary Islands to the mountainous regions of the northern Iberian Peninsula, as well as coastal and insular ecosystems in both the Atlantic and Mediterranean regions. Initially, this study aimed to analyze all 16 parks to provide a comprehensive assessment of vegetation dynamics across the entire network. However, due to limitations in remote sensing data availability, parks located in the Canary Islands were excluded.

**Fig. 1:**
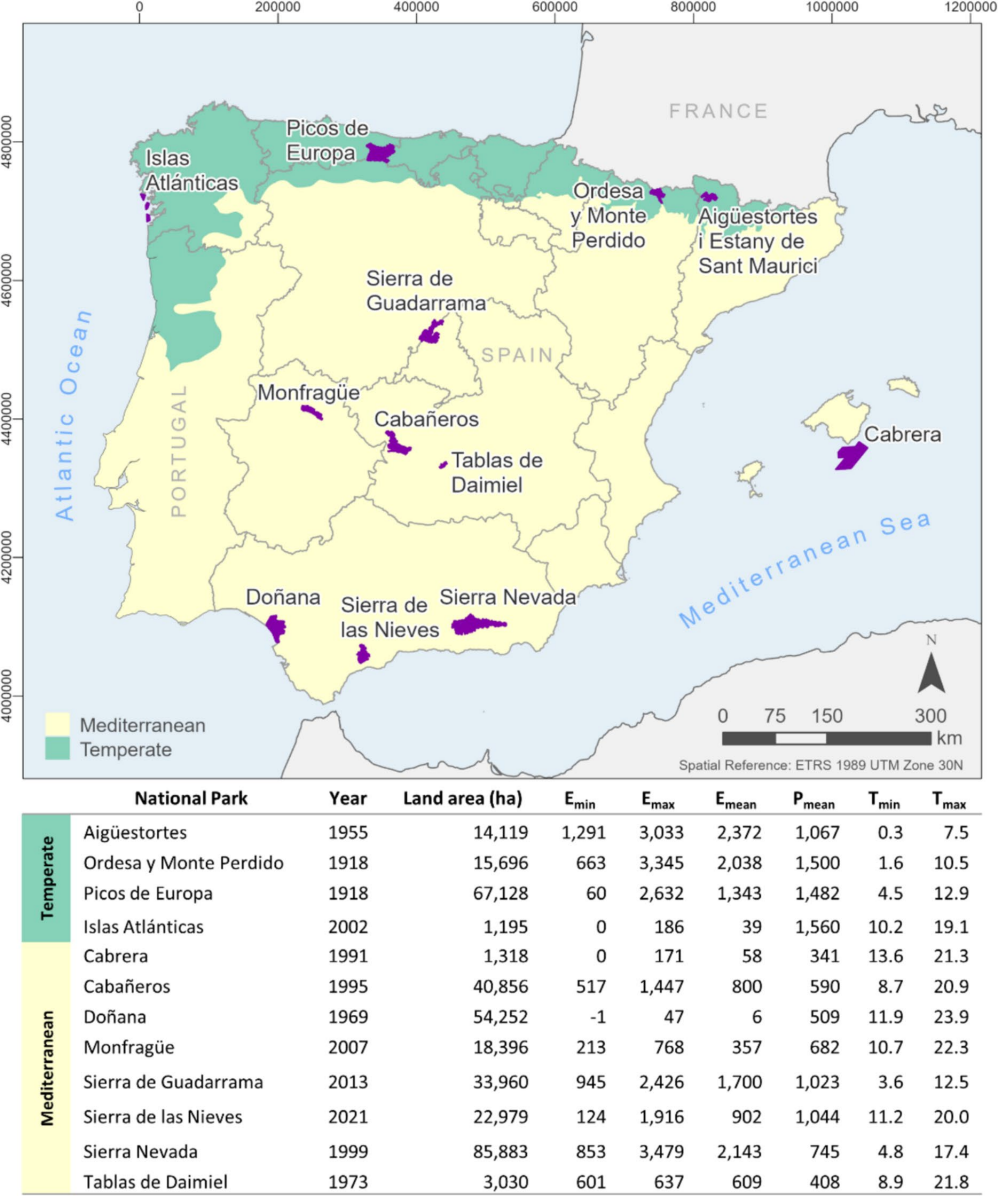
Location of the 12 national parks included in this study and the two biogeographical regions: Temperate (green) and Mediterranean (yellow). The table provides the designation year, land area (ha), minimum (Emin), maximum (Emax), and mean (Emean) elevation (m), mean annual precipitation (Pmean, mm), and mean minimum (Tmin) and maximum (Tmax) temperature (°C) for each park. Elevation data were derived from Spain’s 25-m resolution Digital Terrain Model (MDT25) and climate data from the interpolation of observational data from meteorological stations

As a result, this study focuses on 12 national parks across mainland Spain and the Balearic Islands (Fig. 1), categorized into two biogeographical regions (Dinerstein et al., 2017):

- Temperate (Eurosiberian) parks: Aigüestortes i Estany de Sant Maurici, Ordesa y Monte Perdido, Picos de Europa, and Islas Atlánticas.
- Mediterranean parks: Archipiélago de Cabrera, Cabañeros, Monfragüe, Sierra de Guadarrama, Sierra de las Nieves, Sierra Nevada, Doñana, and Las Tablas de Daimiel.

**Fig. 2:**
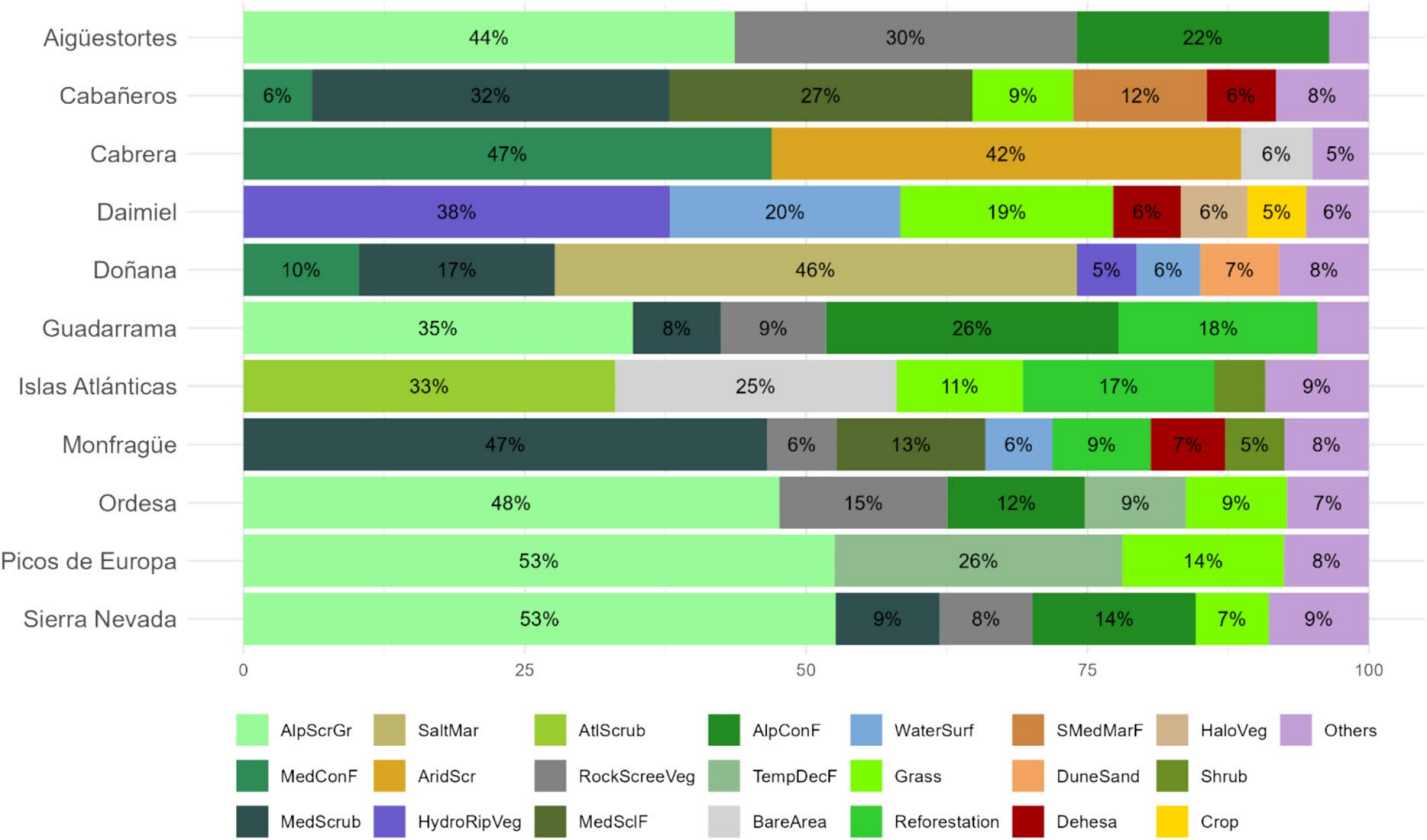
Percentage distribution of natural vegetation systems across 11 Spanish national parks (Sierra de las Nieves cartography is not available). Each bar represents the proportion of various natural systems present within each park, as categorized by specific vegetation types (Table S1). Only categories that occupy more than 5% of the total park area are shown, with the remaining categories combined into an “Others” category. Abbreviations: AlpScrGr, Alpine Scrublands and Grasslands; AlpConF, Alpine coniferous forest; TempDecF, Temperate broadleaf deciduous forest; SMedMarF, Semi-Mediterranean Marcescent Forests; MedConF, Mediterranean coniferous forests; MedSclF, Mediterranean sclerophyllous forest; AtlScrub, Atlantic scrublands; MedScrub, Mediterranean scrublands; AridScr, Hyperxerophilous Garrigues and Scrublands; Grass, Mediterranean and Atlantic grasslands; RockScreeVeg, Rocky and Scree vegetation; BareArea, Bare areas; Reforestation, Reforestation; Dehesa, Dehesas, woody savanna; Crop, Crops; HaloVeg, Halophilous vegetation; HydroRipVeg, Hydrophilous and riparian vegetation; SaltMar, Salt marshes; WaterSurf, Water surfaces

These parks encompass a wide variety of natural vegetation systems, as classified under Spanish Law 30/2014 on National Parks (https://www.miteco.gob.es/es/parques-nacionales-oapn/red-parques-nacionales/sig/sistemas-naturales.html, last accessed January 2025), reflecting the diversity in altitude, climate, and geography across the National Parks network (Fig. 2).

Aigüestortes i Estany de Sant Maurici (hereafter, Aigüestortes), Ordesa y Monte Perdido (hereafter, Ordesa), and Picos de Europa in the northern Iberian Peninsula are characterized by alpine and subalpine coniferous forests, rocky scree vegetation, and extensive grasslands, shaped by their high elevations and cooler climates. These parks receive substantial precipitation, with Ordesa and Picos de Europa averaging 1500 mm annually, while Aigüestortes receives approximately 1000 mm. They also experience lower mean annual temperatures, with average minimum temperatures ranging from 0 to 5 °C and maximum temperatures between 7 and 13 °C.

Doñana and Las Tablas de Daimiel (hereafter, Daimiel), situated in lower-lying Mediterranean regions, are dominated by wetlands, riparian forests, and sclerophyllous vegetation. These parks have significantly warmer climates, with Doñana showing the highest mean maximum temperature (23.9 °C) and an annual rainfall of approximately 500 mm, while Daimiel receives around 400 mm of precipitation per year.

The insular parks, including the Archipiélago de Cabrera (hereafter, Cabrera) in the Mediterranean Sea and Islas Atlánticas in the Atlantic Ocean, represent unique systems that encompass both marine and coastal ecosystems. Cabrera, with an average annual rainfall of 340 mm, hosts distinct hyper-arid vegetation formations alongside Mediterranean sclerophyllous and coniferous forests. In contrast, Islas Atlánticas, which receives around 1500 mm of precipitation annually, is primarily characterized by humid Atlantic scrublands, reflecting its wetter, oceanic climate.

Mediterranean mountain parks such as Sierra Nevada, Sierra de Guadarrama, and Sierra de las Nieves support a diverse mix of sub-Mediterranean and Mediterranean broadleaf forests, Mediterranean coniferous forests, scrublands, and grasslands. At higher elevations, particularly in Sierra Nevada and Sierra de Guadarrama, these parks feature extensive alpine coniferous forests along with alpine scrublands and grasslands. Among them, Sierra de Guadarrama stands out for its colder and wetter climate, with a mean annual precipitation of approximately 1,020 mm and temperatures ranging from 3.6 °C (minimum) to 12.5 °C (maximum). In contrast, Sierra Nevada experiences a slightly warmer and drier climate, with temperatures ranging from 4.8 to 17.4 °C and an annual precipitation of approximately 745 mm. Sierra de las Nieves, located at a lower elevation, presents the highest temperatures of the three, with a mean minimum of 11.2 °C and a mean maximum of 20.0 °C.

### Satellite data preprocessing

To analyze long-term vegetation dynamics across Spain’s national parks, we used Landsat imagery from Landsat 5, 7, and 8. Image preprocessing was carried out in Google Earth Engine (GEE), a cloud-based platform that facilitates the efficient management and analysis of large-scale remote sensing datasets (Gorelick et al., 2017).

A preliminary assessment of image frequency and distribution was conducted to identify potential gaps in the Landsat image collections available in GEE. This analysis involved computing the number of valid images per year for each park, considering only those with less than 80% cloud cover, as indicated in the image metadata (Fig. S1). The results revealed substantial variability in data availability across the national park network (Fig. S2). This issue was particularly pronounced in the Canary Islands’ national parks (i.e., Garajonay, Taburiente, Teide, and Timanfaya), where persistent cloud cover led to significant data gaps. Given these limitations, these parks were excluded from the analysis.

To define the dataset for analysis, we filtered the image collections based on spatial and temporal criteria, selecting only images that intersected the boundaries of the national parks and fell within the study period (1984–2023), ensuring a long-term dataset for vegetation trend analysis. Additionally, since the cloud cover percentage provided in the metadata refers to the entire image rather than the specific area of interest, we computed the actual cloud cover for each image over the park’s land surface. Only images with less than 80% cloud cover within the park boundaries were retained to maintain data reliability.

Once the images were selected, additional preprocessing steps were applied to ensure radiometric consistency across the different Landsat missions. Because spectral responses vary between Landsat 5, 7, and 8, a harmonization process was performed using Reduced Major Axis (RMA) regression coefficients (Roy et al., 2016) to standardize reflectance values. Furthermore, clouds, shadows, and saturated pixels were masked using the “QA_PIXEL” band from Landsat Surface Reflectance Level 2, Tier 1, Collection 2.

Given the diverse topography of Spain’s national parks, additional corrections were necessary to account for illumination differences and terrain-induced variations in reflectance. A Bidirectional Reflectance Distribution Function (BRDF) correction was applied following the methodology described by Poortinga et al. (2019) to standardize reflectance values regardless of sun and sensor viewing angles. Additionally, the modified Sun-Canopy-Sensor with C-correction (SCS + C) topographic correction (Soenen et al., 2005) was applied to mitigate terrain effects, using Spain’s 25-m resolution Digital Terrain Model (MDT25) (https://centrodedescargas.cnig.es/CentroDescargas/index.jsp, last accessed January 2025).

Finally, images were composited into monthly mosaics using the medoid method. This method is preferred over the traditional maximum Normalized Vegetation Index (NDVI) composite due to its ability to produce multi-dimensional median-like values, which are more robust against outliers. It preserves the relationships between the spectral bands, resulting in imagery that better represents the temporal characteristics of the study area (Flood, 2013).

### Vegetation indices calculation

Several vegetation indices were then calculated from the Landsat preprocessed time-series, including the Normalized Difference Vegetation Index (NDVI) (Rouse et al., 1974), Soil-Adjusted Vegetation Index (SAVI) (Huete, 1988), Kernel NDVI (kNDVI) (Camps-Valls et al., 2021), and the Normalized Difference Moisture Index (NDMI) (B. Gao, 1996). These indices were selected for their well-established performance and their ability to capture complementary aspects of vegetation dynamics across a wide range of ecosystems. NDVI serves as a standard measure of greenness and photosynthetic activity by contrasting red absorption and near-infrared (NIR) reflectance, although it is known to saturate in high-biomass areas—particularly in dense forests—where it tends to underestimate productivity (Aklilu Tesfaye & Gessesse Awoke, 2021; S. Gao et al., 2023; Mutanga et al., 2023). To address this limitation, we included kNDVI, which applies a non-linear kernel function to enhance sensitivity to vegetation density variations and mitigates saturation effects in high-biomass regions (Camps-Valls et al., 2021). kNDVI has demonstrated superior performance in vegetation monitoring, showing strong correlations with canopy chlorophyll content (*r* = 0.919–0.933) and improved alignment with flux tower data compared to traditional indices (Q. Wang et al., 2023). SAVI was employed to reduce the influence of soil background, particularly important in drylands and sparsely vegetated areas (Huete, 1988; Qi et al., 1994). This index has been widely validated in arid and semi-arid ecosystems, demonstrating improved accuracy in vegetation monitoring when soil exposure is significant (Bannari et al., 1995; Jinru & Su, 2017). NDMI was included to assess vegetation moisture, a key factor for detecting water stress and drought conditions. This index leverages the contrast between near-infrared (NIR) and shortwave infrared (SWIR) bands to capture variations in canopy water content and has shown high sensitivity both in forested environments (Jin & Sader, 2005; Wilson & Sader, 2002) and arid and semi-arid ecosystems (Malakhov & Tsychuyeva, 2020).

The indices were calculated using the following equations:

1 $$NDVI=\frac{NIR-R}{NIR+R}$$

2 $$SAVI= \frac{\left(NIR-R\right)x\left(1+L\right)}{NIR+R+L}$$

3 $$kNDVI=\mathit{tan}h\left(\frac{{\left(NIR-R\right)}^{2}}{{4\sigma }^{2}}\right)$$

4 $$NDMI=\frac{NIR-SWIR1}{NIR+SWIR1}$$

where NIR is the near-infrared reflectance (Landsat 5/7: Band 4; Landsat 8: Band 5), R is the red reflectance (Landsat 5/7: Band 3; Landsat 8: Band 4), and SWIR1 is the shortwave infrared reflectance (Landsat 5/7: Band 5; Landsat 8: Band 6). The L parameter in SAVI is a soil brightness correction factor, typically set to 0.5 for areas with intermediate vegetation cover. The σ parameter in kNDVI, which controls the sensitivity of the index to variations in vegetation density, was set to 0.15, following Camps-Valls et al. (2021).

The resulting time series of vegetation indices were exported from GEE as GeoTIFF files at a 30 m spatial resolution, with monthly composites covering the period 1984–2023. These files were subsequently converted to NetCDF format in lat-lon WGS84 coordinates for further local analysis. Each NetCDF file contained 480 temporal bands, corresponding to the monthly records over the study period.

### Gap filling

To handle missing data, often caused by cloud cover or satellite data gaps, we applied an interpolation-based gap-filling approach following the methodology of Vicente-Serrano et al. (2025). This method leverages the temporal correlation of vegetation index time series to reconstruct missing values while preserving the statistical properties of the original dataset.

**Fig. 3:**
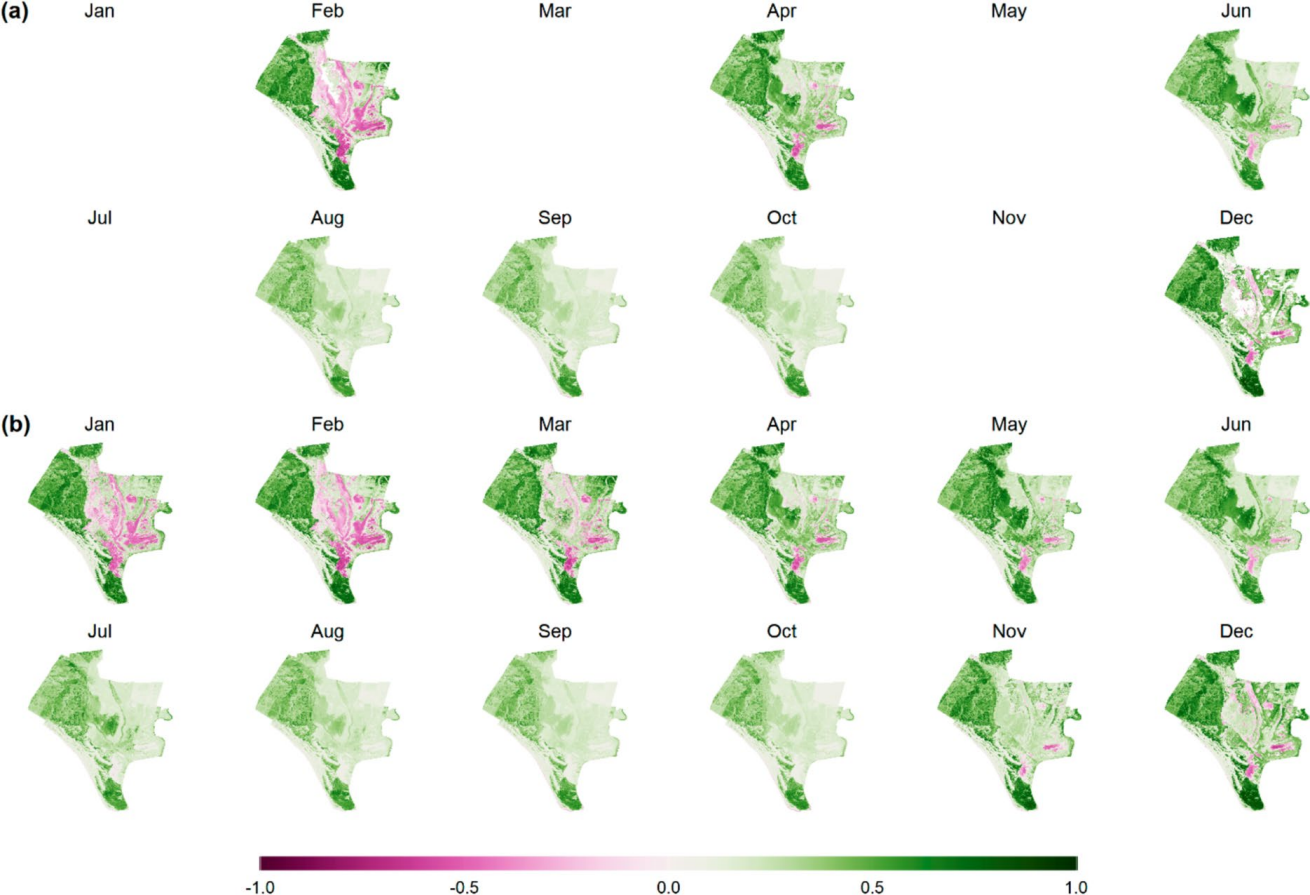
Monthly NDVI (Normalized Difference Vegetation Index) maps of the Doñana National Park for the year 1988. **a** The original NDVI values before gap filling, and (**b**) The NDVI values after applying the gap-filling procedure

The procedure involved dividing the time series into monthly subseries and computing the Empirical Cumulative Distribution Function (ECDF) for each month. Missing values were estimated using the most temporally correlated month within the same annual cycle. This approach ensured that the filled values remained consistent with seasonal patterns while minimizing distortions in long-term trends. Once the missing values were interpolated, the ECDFs were recalculated to maintain the statistical distribution of each monthly subseries. This correction helped preserve the original data structure, ensuring that the imputed values did not introduce biases into subsequent trend analyses. To illustrate the impact of the gap-filling process, Fig. 3 presents the NDVI time series for Doñana National Park in 1988, showing the original dataset before interpolation (Fig. 3a) and the corrected dataset after gap filling (Fig. 3b).

To assess the reliability of the reconstructed NDVI time series, we conducted a pixel-wise evaluation comparing the reconstructed series to the original one. This evaluation included error and agreement metrics (RMSE, R^2^, Willmott’s d) and trend consistency (Theil–Sen slope). Full methodological details and an additional cross-validation analysis—based on artificial gap creation—are provided in Supplementary Material (see Supplementary Method).

### Trend analysis

The long-term time series of vegetation indices were analyzed to detect trends and assess changes in vegetation dynamics over the study period. To identify monotonic trends, we employed the Mann–Kendall test, a non-parametric statistical method widely used for time-series trend detection (Kendall, 1948; Mann, 1945). The Theil-Sen slope estimator (Sen, 1968) was used to quantify the magnitude of trends, providing a robust measure of change resistant to outliers.

Since different vegetation indices (e.g., NDVI, kNDVI, SAVI, and NDMI) have distinct value ranges, direct comparison of trend magnitudes could lead to inconsistencies. To address this, we standardized the Theil-Sen slope values using z-score normalization, which allows for direct comparison across indices (Fig. S3).

This transformation was performed as follows:

5 $$Z= \frac{X-\mu }{\sigma }$$

where *X* represents the raw Theil–Sen slope value, *μ* is the mean slope value for a given index, and *σ* is its standard deviation.

After standardizing the slope values for each park independently, pixels exhibiting significant positive trends were classified into three categories based on their z-score:

- Low: 0.0 ≤ *Z* < 0.5
- Medium: 0.5 ≤ *Z* < 1.5
- High: *Z* ≥ 1.5

Since negative trends were marginal in most of the parks, they were not classified into categories. This classification ensured that differences in the inherent scales of the indices did not affect the interpretation of vegetation trends across the study area.

The final trend analysis outputs included spatially explicit maps of trend magnitude and statistical significance.

### Topographic influence on vegetation trends

Topography plays a critical role in shaping local climatic conditions by influencing solar radiation, temperature, and moisture availability. Three key topography factors—slope, aspect, and elevation—exert significant control over vegetation distribution and dynamics (Liang et al., 2024). To assess how vegetation activity evolved across different topographic settings, we analyzed the distribution of significant vegetation trends based on terrain aspect and bioclimatic zones, which are closely linked to elevation gradients.

Terrain aspect, which determines solar exposure, was derived from the MDT25. Slope aspects were classified into eight directional categories: North (N), Northeast (NE), East (E), Southeast (SE), South (S), Southwest (SW), West (W), and Northwest (NW). To examine the influence of aspect on vegetation trends, we calculated the proportion of pixels with significant positive trends for each aspect category. For a broader interpretation, we grouped aspects into two major classes based on solar exposure:

- North-facing slopes (N_f_): Computed as the mean proportion of significant positive trends across N, NE, and NW slopes.
- South-facing slopes (S_f_): Computed as the mean proportion of significant positive trends across S, SE, and SW slopes.

The percentage of pixels showing significant positive trends was analyzed separately for winter (January) and summer (July) to assess seasonal variations in aspect-related vegetation dynamics. Additionally, we calculated the difference in trend proportions between N_f_ and S_f_ slopes to evaluate how solar exposure influenced vegetation changes across seasons.

To assess the influence of elevation on vegetation dynamics, we examined vegetation trends across bioclimatic zones, as defined in Spain’s vegetation series map (Rivas-Martínez, 1987) (https://www.miteco.gob.es/ca/biodiversidad/servicios/banco-datos-naturaleza/informacion-disponible/memoria_mapa_series_veg_descargas.html, last accessed January 2025). These zones represent a zonation based on the altitudinal thermal gradient, with each zone characterized by distinct vegetation series adapted to specific climatic conditions. The proportion of pixels with significant positive trends was computed for each bioclimatic zone within each park, with separate analyses for winter (January) and summer (July) to assess seasonal differences.

### Vegetation dynamics by natural vegetation systems

Usually, analyses of vegetation dynamics often rely on land-use and land-cover (LULC) change maps to assess the impacts of anthropogenic and natural factors on ecosystems. Among these, the European CORINE Land Cover (CLC) maps have been widely used to evaluate changes in National Parks and protected areas (e.g., Calvache et al., 2016; Kubacka et al., 2022; Martínez-Fernández et al., 2015; Mingarro & Lobo, 2023; Žoncová, 2020). However, CLC maps have significant limitations due to their associated errors and uncertainties (García-Álvarez & Camacho Olmedo, 2017; García-Álvarez et al., 2023). Additionally, the CLC Change (CHA) layers indicate minimal vegetation changes in the study areas, with most detected differences likely arising from interpretative inconsistencies rather than actual land cover changes. Given the absence of high-resolution and reliable change maps for the study period in the National Parks, we analyzed vegetation trends across vegetation types using detailed maps of natural vegetation systems from the Spanish National Park Network (https://www.miteco.gob.es/es/parques-nacionales-oapn/red-parques-nacionales/sig/sistemas-naturales.html, last accessed February 2025). To facilitate the analysis, we primarily used higher hierarchical levels of classification, and in some cases, we aggregated similar categories (Table S1).

The proportion of pixels exhibiting significant positive trends was computed for each natural vegetation system, separately for winter (January) and summer (July), to evaluate seasonal differences in vegetation responses. This analysis provided insights into whether specific vegetation types—such as Alpine coniferous forests, Mediterranean sclerophyllous forest, or riparian vegetation—experienced stronger or weaker long-term greening trends compared to others.

## Results

### Seasonal and annual variability of vegetation indices across Spanish National Parks

**Fig. 4:**
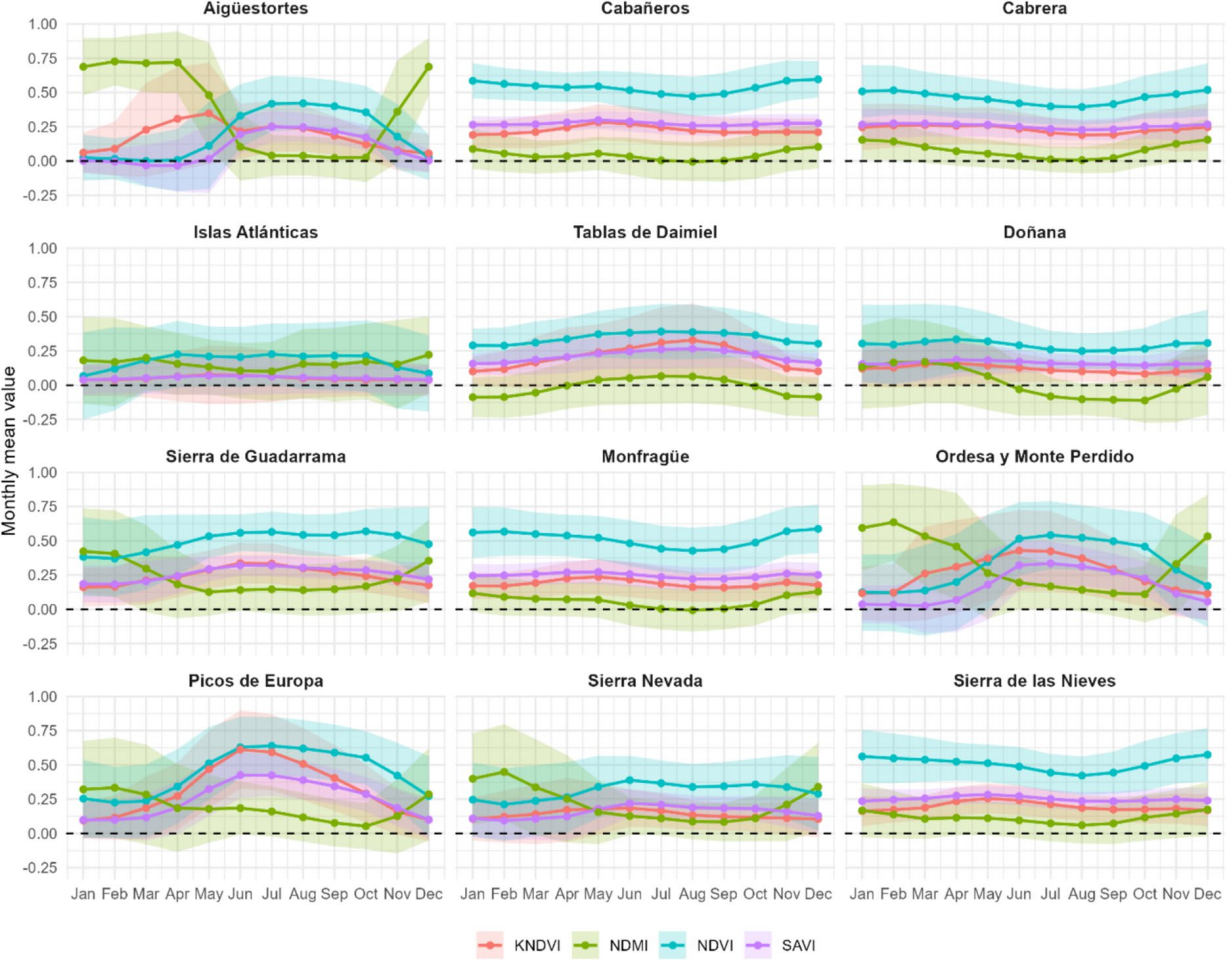
Monthly mean values and standard deviation (shaded areas) of vegetation indices (kNDVI, NDMI, NDVI, and SAVI) for 12 Spanish national parks over a period of 40 years. The x-axis represents months (January to December), and the y-axis indicates the mean index values. Each panel corresponds to a specific national park, with fixed y-axis scales for easier inter-park comparison. Seasonal patterns are evident, with higher index values in spring and summer, reflecting vegetation growth dynamics

Figure 4 illustrates the monthly mean values and standard deviations of vegetation indices (NDVI, kNDVI, NDMI, and SAVI) across the 12 Spanish national parks over the 40-year period. Most indices exhibited clear seasonal patterns, though with regional variations reflecting the climatic and biogeographic diversity of the parks. In Eurosiberian mountain parks, such as Picos de Europa, Aigüestortes, and Ordesa, higher values were recorded in spring and summer (April to August), while lower values occurred in winter (November to February). This pattern reflected the seasonal cycle of temperate ecosystems, where vegetation growth is primarily limited by low temperature and snow cover dynamics. However, the onset of vegetation activity varies among these parks, with SAVI values beginning to increase earlier in Picos de Europa (March) compared to Ordesa (April) and Aigüestortes (May). Conversely, in Mediterranean parks, including Cabañeros, Cabrera, Doñana, and Monfragüe, the opposite trend was observed, with higher values in winter, corresponding to cooler and wetter conditions, and lower values in summer, associated with drought-induced vegetation stress characteristic of the Mediterranean climate, where water availability becomes the main limiting factor for vegetation growth.

Among the indices, NDVI generally showed the highest values, whereas kNDVI closely resembles SAVI, both exhibiting less variability due to their formulations that address saturation and soil background effects, respectively. NDMI effectively captured vegetation and soil moisture, showing lower values during dry months and peaks during wetter periods. Significant differences between parks were also observed. Picos de Europa displayed a greater amplitude in monthly values, likely due to its altitudinal and climatic heterogeneity, while arid parks such as Cabrera showed minimal seasonal variation throughout the year. The wetlands of Daimiel and Doñana were the only parks where NDMI reached negative values, though in different seasons. In Daimiel, negative NDMI values were observed in winter (November–March), while in Doñana, they occurred in summer and early autumn (June–November), indicating distinct seasonal moisture dynamics and hydrological regimes between the two wetland ecosystems.

**Fig. 5:**
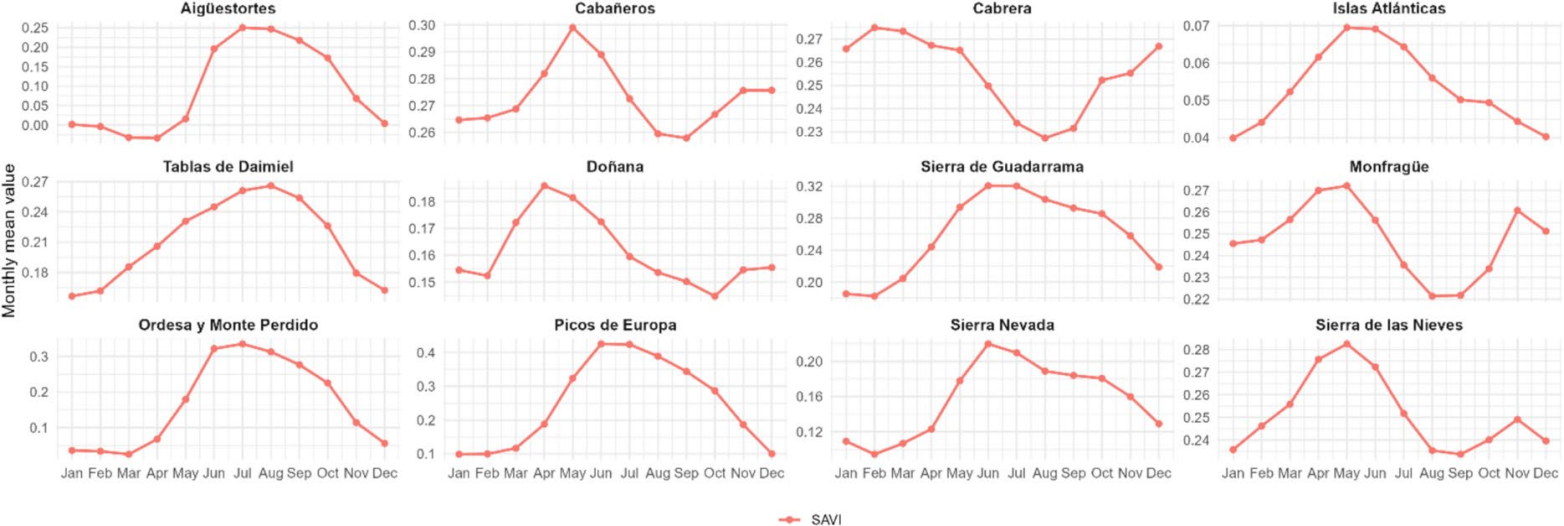
Monthly mean values of the SAVI index for 12 Spanish national parks over a period of 40 years. The x-axis represents months (January to December), and the y-axis indicates the mean SAVI values. Each panel corresponds to a specific national park, with variable y-axis scales to better highlight seasonal vegetation dynamics

While all vegetation indices were analyzed to assess seasonal and long-term trends, SAVI was selected for visualizing and presenting the results because it showed a high correlation with the overall vegetation trends captured by other indices (Figs. S4–S5). Therefore, to enhance clarity and focus on seasonal vegetation dynamics, Fig. 5 presents the monthly mean values of the SAVI index across national parks. By isolating SAVI, which corrects for soil background effects, seasonal patterns of vegetation become more evident. The range of monthly mean values, defined as the difference between the highest and lowest average monthly values (Δavg), was particularly notable in Picos de Europa (Δavg: 0.33), Ordesa (Δavg: 0.31), and Aigüestortes (Δavg: 0.28). These parks exhibited pronounced seasonal variations, with SAVI values fluctuating significantly between periods of vegetation dormancy and active growth. In contrast, parks with low variability, such as Islas Atlánticas, Cabañeros, Cabrera, Doñana, Monfragüe, and Sierra de las Nieves, showed Δavg values below 0.05, indicating relatively stable SAVI values throughout the year, which reflects more uniform climatic conditions or vegetation types. Sierra de Guadarrama and Sierra Nevada showed intermediate seasonal fluctuations, with Δavg values around 0.14. Table S2 presents Δavg values for all the vegetation indices and parks.

### Spatial patterns and magnitude of vegetation trends

**Fig. 6:**
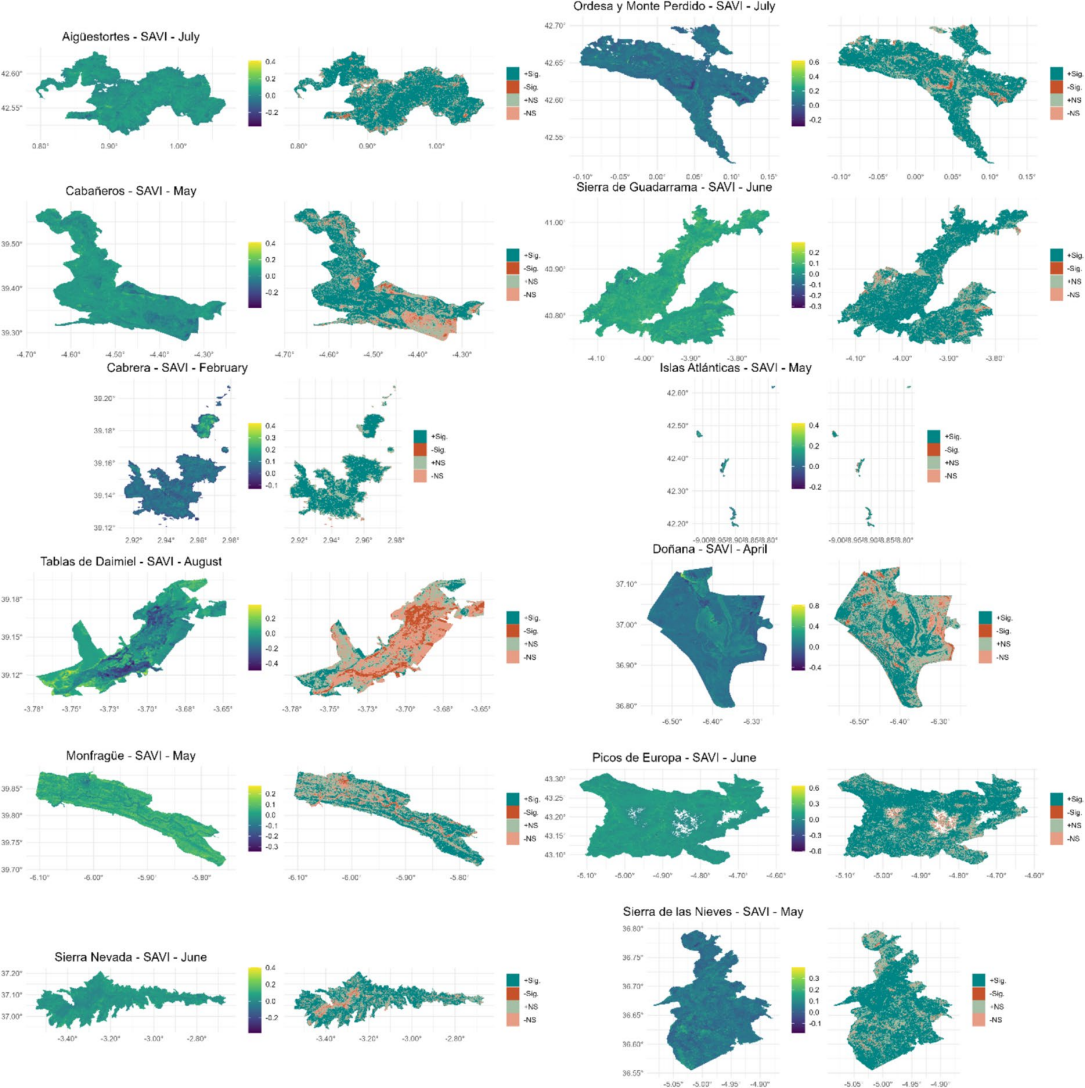
Spatial distribution of vegetation trends based on the SAVI index for the month of peak vegetation activity in 12 Spanish national parks. Each panel displays two maps: (1) the total magnitude of change over the 40-year period (left), represented using a continuous color scale, and (2) the classification of trends (right), showing significant positive trends (+ Sig.), significant negative trends (− Sig.), and non-significant trends (+ NS, − NS). The title of each panel indicates the national park, the vegetation index (SAVI), and the corresponding month. Gaps observed in some areas, particularly in high mountain regions, correspond to pixels with very few observations in the historical series, which could not be filled

Figure 6 presents the spatial distribution of SAVI trends magnitudes and their significance for each national park, focusing on the month of peak vegetation activity—identified as the month with the highest mean SAVI value. This figure highlights the spatial heterogeneity of vegetation trends, with some parks displaying widespread positive trends during their peak activity, while others exhibit mixed or localized patterns of change.

**Fig. 7:**
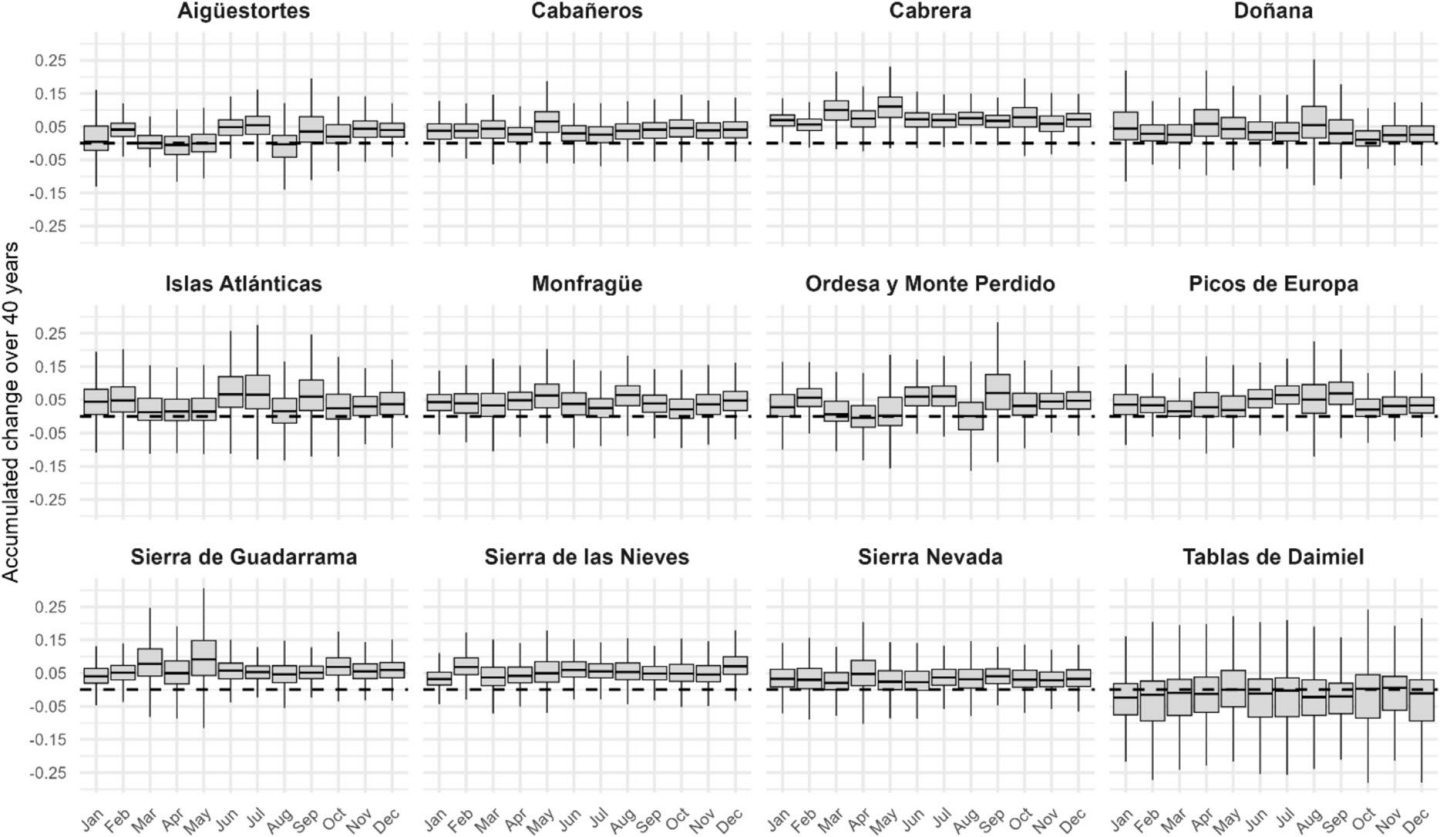
Accumulated change in SAVI over 40 years (1982–2022) across 12 Spanish national parks. The boxplots display the distribution of accumulated changes for each month, calculated using all valid pixels, including both significant and non-significant trends. The y-axis shows the magnitude of the changes, with positive values indicating an increase and negative values a decrease in SAVI. The dashed horizontal line at *y* = 0 represents no change. Scales on the y-axis are adjusted dynamically to each park to better visualize the variability in trends. Outliers are excluded for clarity

Figure 7 presents the monthly distribution of accumulated trends over the 40-year study period, showing the magnitude of changes in SAVI across parks. Table 1 provides percentage changes relative to the mean SAVI values for each month, allowing for a clearer interpretation of long-term trends in vegetation activity.

**Table 1:** Percentage of accumulated SAVI change over 40 years relative to the monthly mean SAVI values. All values are expressed as percentages

National park	Jan	Feb	Mar	Apr	May	Jun	Jul	Aug	Sep	Oct	Nov	Dec
Aigüestortes	− 41.4	132.0	10.3	− 12.3	221.5	27.8	19.4	16.6	18.4	25.3	29.4	16.9
Cabañeros	24.8	10.3	13.9	13.4	13.7	9.0	10.9	14.3	15.8	14.5	16.6	15.8
Cabrera	41.7	27.0	27.6	26.1	25.4	28.0	30.9	24.8	30.9	23.2	30.7	37.5
Islas Atlánticas	34.9	32.6	29.0	71.3	85.3	94.3	102.8	85.2	72.9	59.6	54.6	30.6
Tablas de Daimiel	0.0	− 7.8	− 12.0	− 11.5	− 8.7	− 1.0	− 4.4	− 5.8	− 4.3	2.5	1.7	− 5.9
Doñana	27.8	38.3	31.8	23.8	16.4	17.7	20.8	18.5	17.1	16.9	6.8	16.7
Sierra de Guadarrama	49.4	27.4	22.7	16.6	17.5	16.6	18.0	16.9	20.3	19.4	26.7	35.6
Monfragüe	25.5	19.5	25.0	15.9	14.3	9.7	15.9	17.7	21.4	15.4	7.9	13.0
Ordesa y Monte Perdido	4.3	− 13.7	1.5	40.4	39.2	18.6	17.7	18.0	16.9	19.8	27.5	10.2
Picos de Europa	19.1	27.3	43.4	18.7	21.3	15.1	12.3	8.5	9.6	10.8	10.8	14.8
Sierra Nevada	21.9	50.3	29.3	26.6	22.9	16.8	10.9	15.9	17.6	15.8	19.0	16.0
Sierra de las Nieves	21.1	17.0	20.9	11.6	17.3	20.3	23.7	29.2	30.2	19.1	19.6	15.5

Islas Atlánticas showed the highest relative increases, particularly between June and July, where changes exceed 90%, peaking at 103% in July. Aigüestortes exhibited exceptional changes in May (222%), corresponding to the onset of vegetative growth following winter dormancy—a pattern characteristic of high-altitude ecosystems. In contrast, Cabañeros, Monfragüe, Cabrera, and Doñana displayed relatively stable positive trends throughout the year, with percentages generally ranging between 10 and 30%. Daimiel showed predominantly negative changes across most months, indicating a decline in vegetation productivity. These spatial patterns set the stage for further analysis of the extent and distribution of significant trends, explored in the following sections.

### Extent and temporal variability of significant trends

**Fig. 8:**
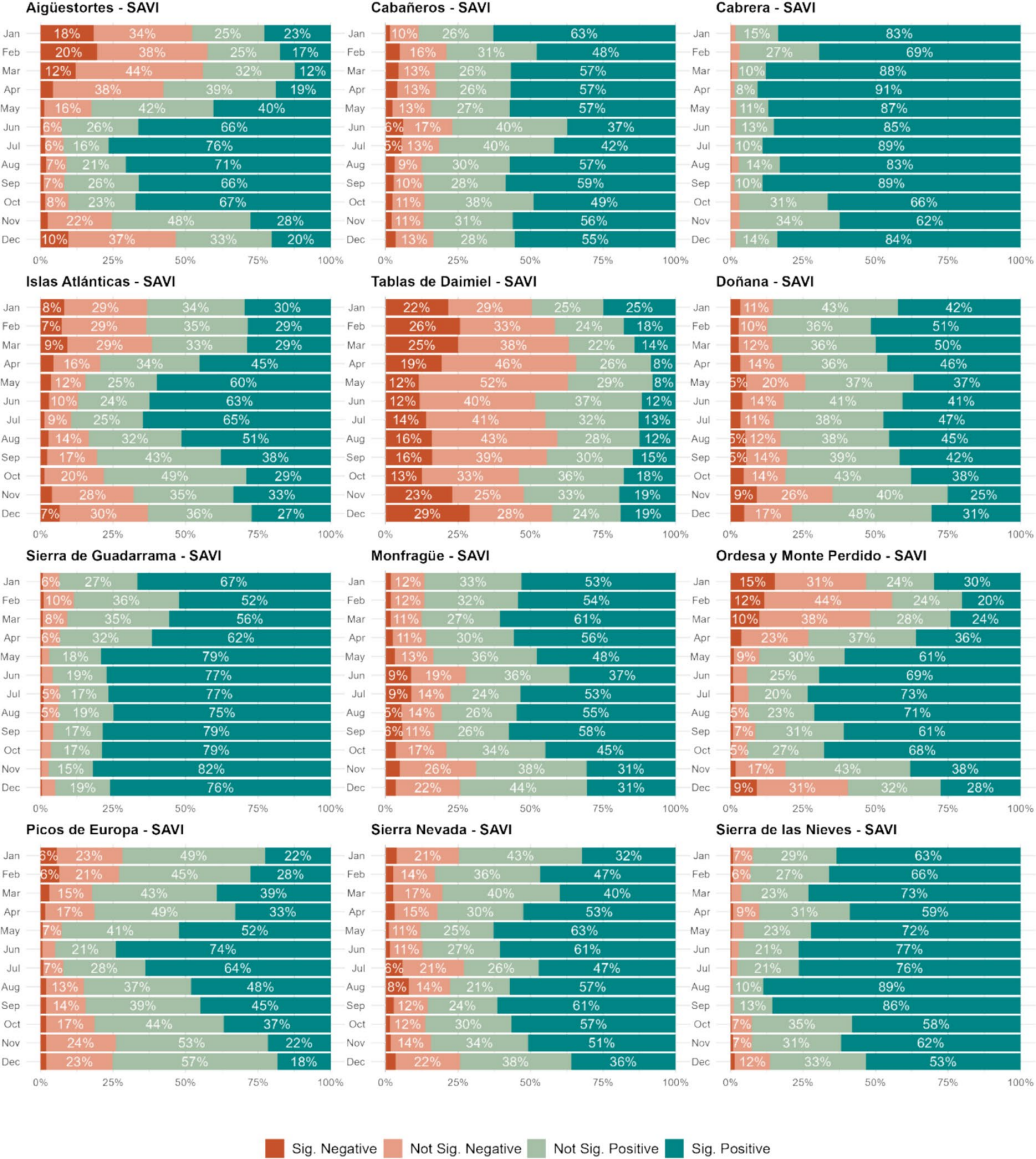
Monthly distribution of significant and non-significant SAVI trends across 12 Spanish national parks. Each bar shows the percentage of pixels within each park for a given month categorized as follows: significant positive trends (dark green), non-significant positive trends (light green), non-significant negative trends (light red), and significant negative trends (dark red). The trends are calculated for each park individually, showing seasonal and spatial variability in vegetation dynamics

**Fig. 9:**
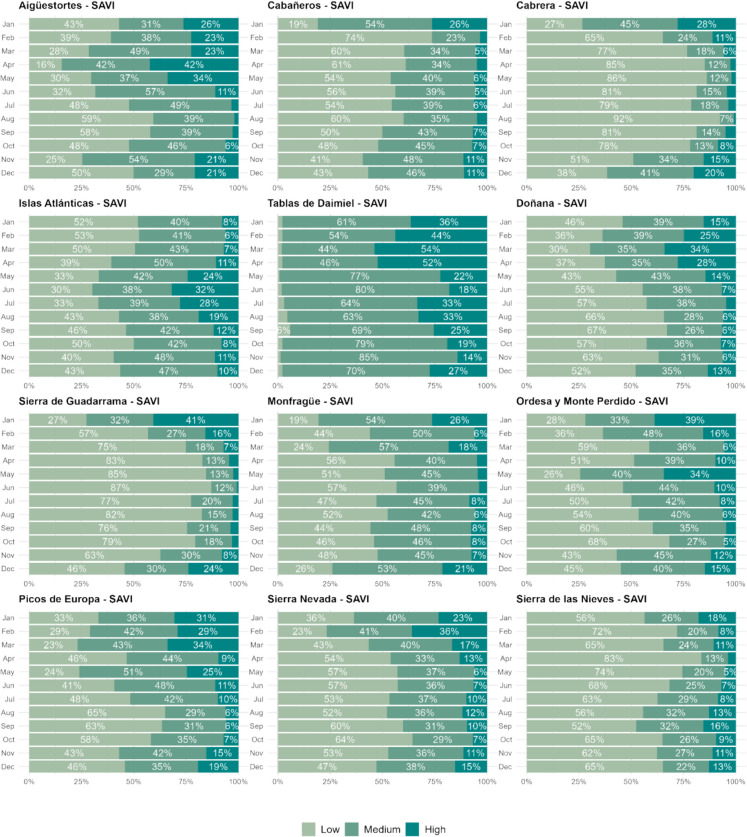
Distribution of significant positive vegetation trends classified by magnitude of change (SAVI) across 12 Spanish national parks on a monthly basis. The magnitude of change is expressed as the total slope change over 40 years, classified into three categories: low (Z-score < 0.5), medium (0.5 ≤ Z-score < 1.5), and high (Z-score ≥ 1.5). Each bar represents the proportion of significant positive trends for each month, showing how the strength of positive trends varies throughout the year across different parks

Figure 8 presents the monthly distribution of significant and non-significant SAVI trends across the 12 national parks, and Fig. 9 presents the distribution of significant positive vegetation trends classified by magnitude of change. The proportion of pixels with significant trends varied by park and season, revealing distinct temporal patterns. Most parks showed a predominance of positive trends throughout the year, indicating an overall increase in vegetation activity. However, the extent of significant trends fluctuated, with the highest proportions occurring during specific months.

Mountainous parks, such as Aigüestortes, Ordesa, and Picos de Europa, exhibited strong seasonal patterns. During winter (November–March), most pixels showed non-significant trends, reflecting the dormant state of vegetation. In contrast, from June to August, over 70% of the pixels in these parks displayed significant positive trends, indicating an overall increase in vegetation activity during the growing season. In these areas, the magnitude of significant trends was generally low to moderate, with 30–40% of pixels falling within the medium trend category (z-score: 0.5–1.5), while large-magnitude trends (z-score > 1.5) were less frequent in summer than in winter.

Mediterranean parks, including Cabrera, Sierra de Guadarrama, and Sierra de las Nieves, displayed a more consistent distribution of positive trends throughout the year. Cabrera, in particular, had the highest proportion of significant positive trends, exceeding 90% in some months. Parks like Cabañeros and Monfragüe showed intermediate percentages, with about 50% of their surface exhibiting significant trends year-round. In these Mediterranean parks, low-magnitude trends were the most frequent, while large-magnitude trends were more common during winter months.

Doñana and Daimiel showed contrasting patterns. Doñana was dominated by positive trends, although the magnitude of changes remained moderate, with only 5–15% of pixels displaying large changes. Daimiel, however, exhibited a concerning pattern, with a high proportion of negative trends (12–29%) in most months, often exceeding the percentage of positive trends. Notably, the significant trends in Daimiel were primarily of medium to large magnitude, while low-magnitude trends were negligible. However, this applied exclusively to areas experiencing positive trends, which were relatively scarce in this park.

Moreover, when analyzing NDMI trends (Fig. S8), both Doñana and Daimiel stood out as the parks with the highest proportion of negative trends. Doñana, in particular, showed a higher percentage of its surface affected by negative trends across most months for this index. Since NDMI index primarily measures vegetation moisture content rather than vegetation activity, this pattern suggests a potential decline in water availability or increased vegetation stress, particularly in these two parks.

These results highlight substantial spatial and temporal variability in vegetation dynamics across Spain’s national parks. While many parks showed evidence of long-term greening, others, such as Daimiel, exhibited worrying declines in vegetation productivity. Additional figures showing the trends and classification of magnitudes for the other vegetation indices (NDVI, kNDVI, and NDMI) are available in the supplementary material for further analysis (Figs. S6-S11).

### Influence of terrain aspect on vegetation trends

Table 2 provides the proportion of significant positive trends observed in north-facing slopes (N_f_: average of N, NE, and NW aspects) and south-facing slopes (S_f_: average of S, SE, and SW aspects).

**Table 2:** Summary of aspect-based vegetation trends in 10 Spanish national parks during January and July. The table presents the average proportion of significant positive trends on North-facing slopes (N_f_, calculated as the mean of N, NE, and NW) and South-facing slopes (S_f_, calculated as the mean of S, SE, and SW), alongside the difference between them (Diff. N_f_-S_f_). It also includes the maximum (Max) and minimum (Min) proportions of significant positive trends, with their corresponding aspect in parentheses, as well as the range of variability across all aspects (Diff. Max–Min)

National park	Month	N_f_ (%)	S_f_ (%)	Diff. N_f_-S_f_ (%)	Max (%)	Min (%)	Diff. Max–Min (%)
Aigüestortes	January	30	16	13	35 (NW)	14 (E)	21
	July	77	75	3	78 (E)	73 (S)	5
Cabañeros	January	68	58	10	74 (W)	52 (S)	22
	July	41	42	− 1	45 (E)	37 (NW)	8
Cabrera	January	91	90	0	93 (E)	88 (NW)	5
	July	97	91	6	98 (N)	90 (S)	8
Islas Atlánticas	January	24	38	− 14	42 (S)	16 (N)	25
	July	70	73	− 2	73 (SE)	68 (W)	5
Monfragüe	January	53	51	2	61 (SW)	42 (E)	19
	July	53	53	0	61 (NE)	40 (NW)	21
Ordesa y Monte Perdido	January	24	31	− 7	35 (SW)	22 (NE)	12
	July	70	75	− 4	76 (S)	68 (N)	8
Picos de Europa	January	16	27	− 11	29 (S)	13 (N)	17
	July	62	66	− 4	68 (S)	60 (N)	8
Sierra Nevada	January	26	36	− 10	38 (SW)	22 (N)	16
	July	49	46	3	52 (NE)	40 (W)	12
Sierra de Guadarrama	January	65	64	1	69 (SW)	61 (S)	7
	July	74	78	− 3	82 (SE)	69 (NW)	12
Sierra de las Nieves	January	61	63	− 2	71 (W)	56 (N)	15
	July	76	77	− 1	77 (NE)	73 (NW)	4

In general, during winter, N_f_ slopes exhibited lower proportions of significant positive trends than S_f_ slopes in mountainous parks, except in Aigüestortes. In Picos de Europa (N_f_: 16%, S_f_: 27%) and Sierra Nevada (N_f_: 26%, S_f_: 36%), S_f_ slopes showed a higher prevalence of positive trends, consistent with the influence of solar radiation exposure. The largest contrast occurred in Islas Atlánticas, where S_f_ slopes exceeded N_f_ slopes by 14 percentage points (N_f_: 24%, S_f_: 38%), followed by Picos de Europa (−11%) and Sierra Nevada (−10%), reinforcing the pattern of stronger vegetation trends on S_f_ slopes during winter. However, Aigüestortes showed the opposite pattern, with higher positive trends on N_f_ slopes (N_f_: 30%, Sf: 16%).

In summer, aspect-related differences are generally less pronounced, with N_f_ and S_f_ slopes showing more similar proportions of positive vegetation trends. Cabañeros, Islas Atlánticas, Monfragüe, Sierra de las Nieves, and Sierra Nevada exhibited minimal differences (< 3%) between N_f_ and S_f_ slopes. The most notable exception was Cabrera, where N_f_ slopes (97%) exceeded S_f_ slopes (91%) by 6 percentage points, making it the only park with a substantial difference in positive trend proportions between these two aspect categories during this season. Aigüestortes also maintained an advantage on N_f_ slopes in summer (3%), while Ordesa (−4%) and Picos de Europa (− 4%) continued to show slightly higher positive trends on S_f_ slopes, though with smaller differences compared to winter.

### Influence of elevation on vegetation trends

**Fig. 10:**
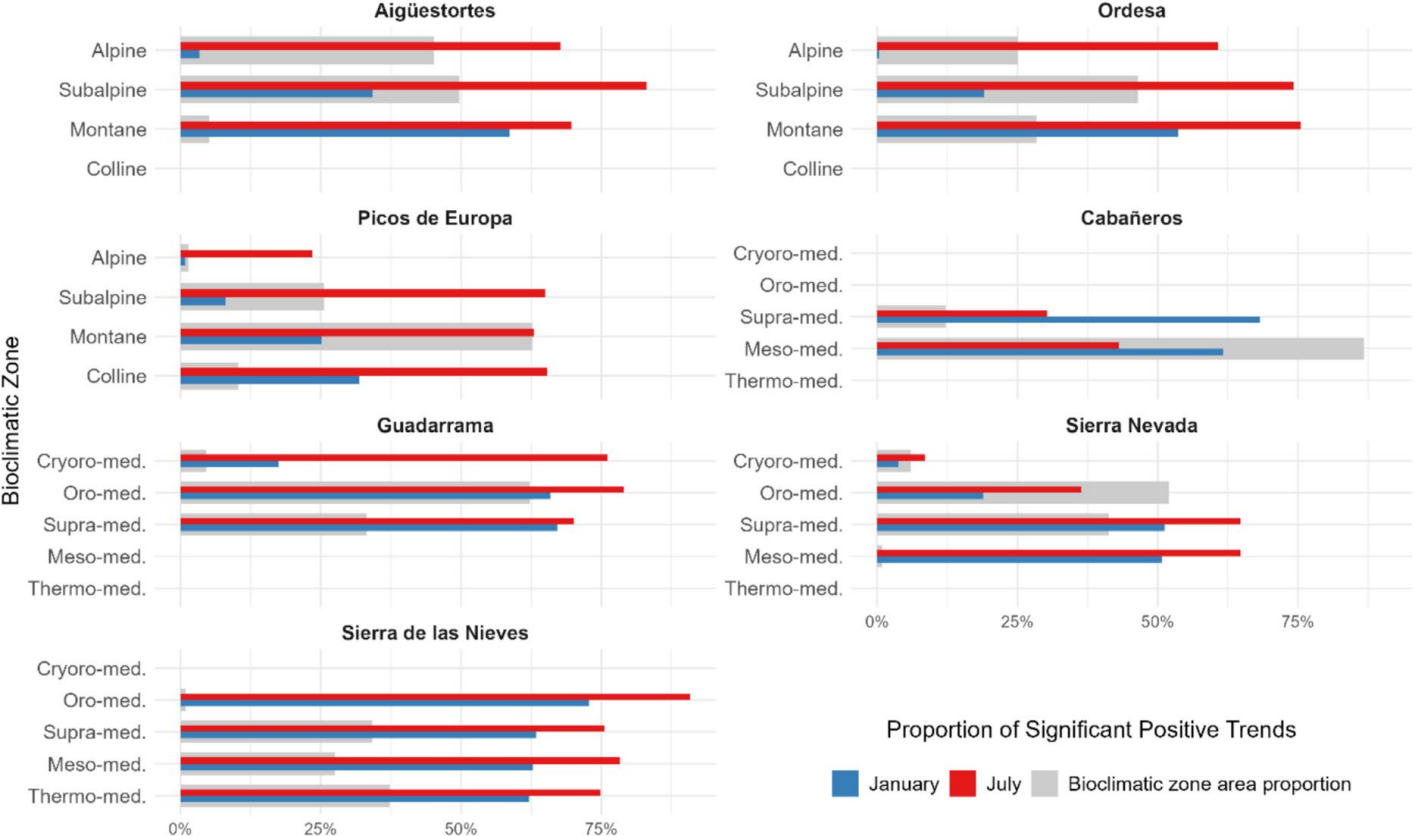
Proportion of significant positive SAVI trends by bioclimatic zone across seven Spanish national parks. Gray bars represent the proportional area of each bioclimatic zone within each park, while the colored bars indicate the percentage of pixels with significant positive trends in January (blue) and July (red). Only parks with at least two bioclimatic zones are included

Figure 10 presents the proportion of pixels with significant positive SAVI trends across bioclimatic zones in seven Spanish national parks. In mountainous parks of the Eurosiberian region, the proportion of significant positive trends varied across elevation gradients, particularly in winter. In Aigüestortes, Ordesa, and Picos de Europa, the proportion of pixels with significant trends decreased with elevation during January, whereas in July, significant trends were more evenly distributed across all bioclimatic zones. This pattern was less pronounced in Sierra de Guadarrama and Sierra Nevada, and was not observed in Sierra de las Nieves, where the proportion of significant trends increased slightly with elevation in both seasons.

Additionally, Sierra de las Nieves showed consistently higher proportions of positive trends across all bioclimatic zones (> 62% in January and > 75% in July) compared to Sierra Nevada, where the proportion remained below 51% in January and below 65% in July. However, it is important to note that the Oro–Mediterranean zone, which dominates Sierra Nevada and Guadarrama, is almost absent in Sierra de las Nieves, and the Cryoro–Mediterranean zone, present in the former two parks, is completely absent in the latter. Cabañeros, characterized by a Mediterranean climate and only two differentiated bioclimatic zones, showed a distinct pattern, with higher proportions of significant trends in winter than in summer. This trend was not observed in other parks and aligns with the patterns found in the aspect analysis (Sect. 3.4).

### Vegetation trends by natural vegetation types

**Fig. 11:**
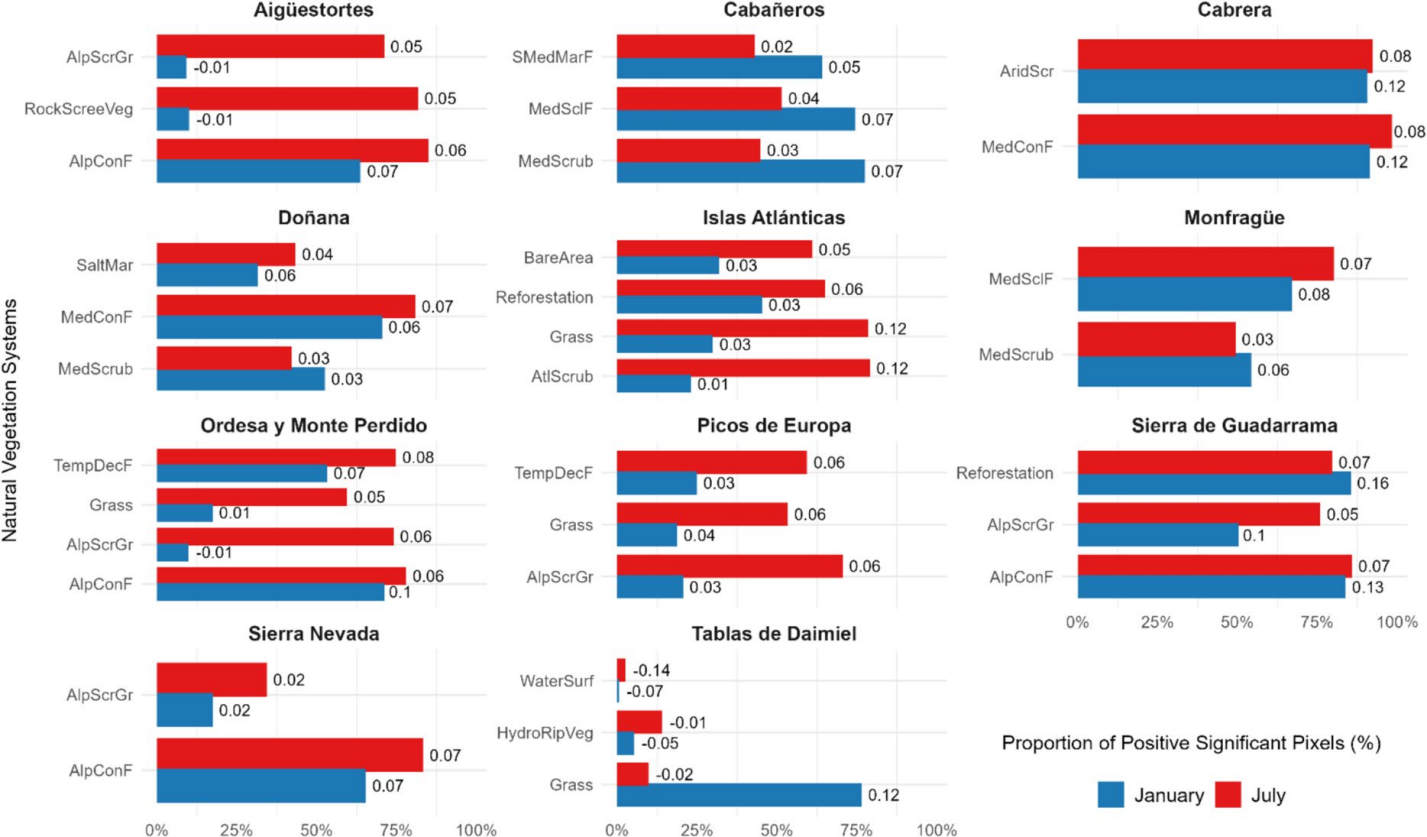
Trends in the SAVI vegetation index. The figure shows the proportion of positive significant pixels (%) across natural vegetation systems for each national park during January (blue) and July (red). Each bar represents the percentage of pixels within a specific vegetation category exhibiting positive significant trends. The numbers on the bars indicate the mean slope of change over the entire study period for each vegetation system and month. Abbreviations: AlpScrGr, Alpine Scrublands and Grasslands; AlpConF, Alpine coniferous forest; TempDecF, Temperate broadleaf deciduous forest; SMedMarF, Semi-Mediterranean Marcescent Forests; MedConF, Mediterranean coniferous forests; MedSclF, Mediterranean sclerophyllous forest; AtlScrub, Atlantic scrublands; MedScrub, Mediterranean scrublands; AridScr, Hyperxerophilous Garrigues and Scrublands; Grass, Mediterranean and Atlantic grasslands; RockScreeVeg, Rocky and Scree vegetation; BareArea, Bare areas; Reforestation, Reforestation; HydroRipVeg, Hydrophilous and riparian vegetation; SaltMar, Salt marshes; WaterSurf, Water surfaces. Only vegetation categories covering more than 10% of the national park surface are included

The trend analysis across natural vegetation systems in the national parks revealed a predominance of significant positive trends in most vegetation categories, suggesting an overall increase or recovery in vegetation activity during the study period (Fig. 11). Notably, Alpine Coniferous Forests (AlpConF) and Mediterranean Coniferous Forests (MedConF) consistently showed high proportions of pixels with significant positive trends, often exceeding 60% throughout the year. This pattern was particularly evident in Aigüestortes and Sierra Nevada, where values surpassed 80% in summer, and in Sierra de Guadarrama and Cabrera, where values remained above 80% and 90%, respectively, for most months of the year. Similarly, Alpine Scrublands and Grasslands (AlpScrGr), dominant in high-altitudinal zones, showed high proportions of positive trends during summer months (> 70%) and accumulated mean magnitude changes ranging between 0.05 and 0.06 over the 40-year period. However, this pattern was not observed in Sierra Nevada, where AlpScrGr, which covers 53% of the park’s surface, exhibited a lower proportion of positive trends (< 34%), and a mean magnitude change of only 0.02.

Vegetation categories closely associated with water availability, such as Hydrophilous and Riparian Vegetation (HydroRipVeg), exhibited the highest proportions of negative trends. This pattern was particularly evident in Daimiel, where negative trends frequently exceeded 30% from November to April and surpassed 60% for water surfaces, highlighting the potential vulnerability of these ecosystems during the study period.

These results highlight the variability of vegetation trends across different ecosystem types, with forests and scrublands generally exhibiting positive trends, while wetland-related vegetation showed more pronounced negative trends, particularly in Daimiel.

## Discussion

This study provides a long-term assessment of vegetation trends across Spain’s National Park Network, leveraging Landsat time series (1984–2023) and Google Earth Engine for efficient data processing. The overall predominance of positive vegetation trends suggests increasing vegetation productivity across most parks, aligning with previous studies that have documented widespread greening in Iberian national parks (Alcaraz-Segura, Cabello, et al., 2008a, 2008b) and broader Mediterranean and Temperate regions (Adell-Michavila et al., 2024; Gutiérrez-Hernández, 2022; Gutiérrez-Hernández & García, 2024; Pausas & Millán, 2019; S.M. Vicente-Serrano et al., 2004, 2020).

Multiple studies have documented the impact of climate change and land-use shifts in high-mountain ecosystems, leading to significant transformations in vegetation structure and composition. These changes often result from the combined effects of climate-driven processes and rural abandonment, which have facilitated forest expansion, shrub densification, and shifts in plant community composition. Examples of these processes have been observed in Picos de Europa, where Hernández-Romero et al. (2022) reported a 23% increase in forest cover between 1989 and 2019, largely attributed to rural abandonment and the subsequent expansion of forests into former pasturelands. Similarly, forest expansion to higher elevations, primarily linked to rural abandonment, have been documented in eastern Pyrenees (Ameztegui et al., 2016). In Sierra de Guadarrama, García-Romero et al. (2010) found that rising temperatures, shorter snow cover duration, and changes in precipitation patterns have favored the expansion of leguminous shrubs, often at the expense of herbaceous vegetation in high-altitude zones. Additionally, the decline of traditional land uses has further contributed to forest densification in mid-elevation zones of Sierra de Guadarrama (Tellería, 2020) and other mountainous parks in the Eurosiberian region, including Picos de Europa, Ordesa, and Aigüestortes (Ameztegui et al., 2021).

Interestingly, Alcaraz-Segura et al. (2008b) reported negative NDVI trends in alpine coniferous forests across these mountainous environments, suggesting a decline in vegetation productivity during their study period (1981–2003). However, our results indicated strong positive trends of high magnitude for these forest types (Fig. 11). This discrepancy may reflect recent climatic conditions favoring tree growth, reduced human disturbance, or methodological differences, particularly the lower (8 km) resolution of AVHRR data used in their study compared to the 30 m Landsat data used here. Additionally, their annual-scale analysis may have masked seasonal variations that are better captured in our monthly trend assessment, particularly in highly seasonal high-mountain ecosystems.

Encroachment of alpine grasslands, driven by the expansion of generalist woody species and the decline of alpine grassland specialists, has been documented in response to land abandonment in Sierra de Guadarrama (Jimenez-Alfaro et al., 2014) and reduced grazing pressure in Ordesa (Alados et al., 2011). Similar trends have been reported in other Mediterranean mountain systems, likely driven by climate warming and changes in disturbance regimes (Sanz-Elorza et al., 2003). This could explain the higher proportions of significant positive trends observed in our study for the Alpine Shrublands and Grasslands (AlpScrGr) category (Fig. 11), particularly in Aigüestortes, Ordesa, and Picos de Europa, and to a lesser extent in Sierra Nevada, where shrub expansion may be contributing to the overall greening patterns.

Vegetation formations characteristic of strictly Mediterranean environments, such as sclerophyllous shrublands, forests, and Mediterranean coniferous woodlands, also exhibited widespread positive trends across parks such as Monfragüe, Cabañeros, and Cabrera, and even in Doñana and Daimiel, where wetland-associated plant communities showed substantial declines. A noteworthy case was Cabañeros, where a higher proportion of the surface exhibited positive trends in winter compared to summer, both for sclerophyllous shrublands and forests as well as sub-Mediterranean marcescent forests (Fig. 11). While this pattern was subtle in the sclerophyllous shrublands of Monfragüe, in Cabañeros, it was particularly pronounced. According to Alcaraz-Segura et al., (2008a, 2008b), this trend is likely driven by rising winter temperatures, which may enhance vegetation productivity during the colder months.

As a representative of coastal Mediterranean ecosystems, Cabrera National Park stood out as the park with the highest proportion of significant positive vegetation trends, with over 90% of its surface showing increases in vegetation indices. These trends align with long-term vegetation recovery following the removal of domestic herbivores and reduced human land use (Rita et al., 2020). Historically, intensive grazing and agricultural activities led to severe vegetation degradation, particularly affecting forest and shrubland communities. However, after Cabrera was designated a National Park in 1991, conservation measures—including the eradication of domestic goats and sheep—allowed for natural forest recovery, leading to an increase in woody vegetation, particularly *Pinus halepensis* and *Juniperus phoenicea* woodlands.

Remarkably, the areas exhibiting the greatest magnitude of change corresponded to the “illa dels Conills” (Fig. 6), the second-largest islet of the Archipelago, where the successful eradication of rats and rabbits (McMinn & Rodríguez, 2010) has likely contributed to vegetation recovery. Similar patterns have been observed in other Mediterranean islands, where removal of herbivores has led to shrubland and forest expansion (Oro et al., 2022).

The study by Rita et al. (2020) also highlights the role of slope orientation in shaping vegetation distribution and density, supporting our findings that north-facing slopes generally show higher proportions of positive trends compared to south-facing slopes in Cabrera (Table 2). Despite Cabrera’s relatively low-relief topography, slope orientation plays a key role in vegetation dynamics. Notably, during summer, Cabrera exhibited the largest difference between north-facing and south-facing slopes (Diff. Nf-Sf = 6%, Table 2) among the national parks analyzed. This highlights the importance of local-scale environmental gradients imposed by slope aspect, particularly in arid ecosystems, where topography has been shown to exert a significantly stronger influence on vegetation dynamics than in temperate ecosystems (Liang et al., 2024).

Unlike the widespread positive trends observed across most parks, Daimiel presented a contrasting scenario, with extensive areas showing negative vegetation trends, particularly in riparian vegetation, which constitutes its primary ecosystem. These patterns are consistent with studies documenting long-term environmental degradation driven by groundwater overexploitation, declining water quality from agricultural runoff and urban wastewater, and periods of extreme drought (Alvarez-Cobelas et al., 2001; Angeler & Sanchez-Carrillo, 2010; Pena-Regueiro et al., 2023; Villar-Argaiz et al., 2022). Meanwhile, Doñana presented predominately positive trends across all vegetation types, including marshlands, contrasting with the negative NDVI trends reported by Alcaraz-Segura et al., (2008a, 2008b). As previously discussed for alpine forests, this discrepancy likely reflects differences in study periods, data resolution, and annual vs. seasonal analysis approaches. However, while SAVI, NDVI, and kNDVI suggest increasing vegetation activity in Doñana, NDMI exhibited negative trends, similar to or even more pronounced than those observed in Daimiel. Since NDMI primarily reflects vegetation moisture rather than productivity, these findings may indicate a transition towards more drought-adapted vegetation, possibly indicating a shift from hygrophytic to xerophytic plant communities. This aligns with recent studies reporting a progressive desiccation of Doñana’s temporary lagoons, driven by groundwater overexploitation and reduced water availability (de Felipe et al., 2023). These patterns suggest that reduced water availability may be reshaping wetland plant communities, favoring species with higher drought tolerance over those adapted to wetter conditions.

The seasonal dynamics of the NDMI index further reinforce the contrasting hydrological regimes between the two wetland parks. In Daimiel, NDMI values remain negative from November to March and turn positive in summer, whereas in Doñana, NDMI peaks in winter and declines in summer (Fig. S12). These differences align with the distinct hydrological systems described by Miguel and Malte (2014): Doñana functions as a surface water–dominated wetland with a highly variable hydroperiod, primarily influenced by seasonal precipitation and evaporation (Florencio, 2010), while Daimiel relies on groundwater inputs, which have been severely disrupted by aquifer overexploitation (Castaño et al., 2018). As a result, Daimiel has become increasingly dependent on artificial water transfers, raising concerns about its long-term sustainability.

Given these findings, future research should explore how vegetation in these contrasting wetland systems will continue to respond to climate change and anthropogenic pressures. Understanding whether these trends indicate a shift in species composition, changes in water availability, or long-term ecological transitions will be critical for informing conservation and water management strategies.

While this study provides a comprehensive analysis of vegetation trends across Spain’s national parks, certain limitations must be acknowledged. Specifically, while we discuss potential drivers of the observed trends in light of existing literature, we do not conduct a formal analysis to quantitatively establish explanatory factors. Instead, our findings underscore the value of remote sensing in monitoring vegetation dynamics, providing a valuable resource for evaluating conservation outcomes. By offering a long-term perspective, this study supports ongoing and future assessments of management interventions undertaken by park administrators and contributes to the identification and refinement of adaptive conservation strategies.

Furthermore, it is important to note that positive vegetation trends identified through satellite derived indices, do not inherently indicate ecological improvement. Vegetation indices are highly effective at capturing changes in vegetation activity and cover but do not provide information to fully characterize the structure, composition, or quality of the vegetation. For instance, the expansion of invasive species or other vegetation shifts driven by anthropogenic pressures may result in positive trends while simultaneously leading to declines in native biodiversity or habitat homogenization (e.g., Landmann et al., 2020; Miller et al., 2021; Tellería, 2020). To fully understand the ecological implications of vegetation changes captured by means of remote sensing, future research should aim to integrate vegetation indices with complementary datasets, such as field-based biodiversity assessments, ecological monitoring, or very high-resolution remote sensing data and methods capable of identifying species-level or structural changes.

## Conclusions

This study has demonstrated the usefulness and potential of Landsat data, processed through Google Earth Engine, for capturing and analyzing vegetation dynamics in Spain’s National Park Network over the past four decades. The main findings include the following:

- Most Spanish’s national parks showed significant positive vegetation trends for the last 40-years period (1984–2023), reflecting forest expansion, densification, and encroachment processes. These trends are likely driven by a combination of rural abandonment, climate warming and management practices.
- High-mountain environments, particularly in temperate regions, exhibited strong seasonality in vegetation trends, with significant increases during the months of active vegetation growth. In contrast, Mediterranean parks showed consistently high proportions of positive trends throughout the year. A singular pattern was observed in Cabañeros, where winter months exhibited higher proportions of positive trends than summer months, likely linked to increasing winter temperatures.
- Cabrera National Park exhibited the highest proportion of positive trends, reflecting shrubland and forest densification in response to land-use changes since its designation and the removal of herbivores, a practice that has proven highly effective in promoting vegetation recovery.
- Tablas de Daimiel experienced widespread declines in vegetation productivity and moisture (NDMI) affecting vegetation formations linked to aquatic ecosystems. Strong evidence indicates that this decline is driven by groundwater depletion and hydrological stress. In contrast, Doñana’s wetlands exhibited mixed trends, with positive SAVI but declining NDMI, suggesting a shift toward drought-tolerant species in response to reduced water availability.
- Aspect-driven dynamics emerge as a key local-scale factor influencing vegetation trends, particularly in arid environments. Differences in solar radiation exposure affect vegetation activity, with north-facing slopes generally showing a higher proportion of positive trends. This pattern is especially relevant in Mediterranean and semi-arid ecosystems, where aspect plays a crucial role in vegetation structure and recovery as we observed in Cabrera.
- Discrepancies between this study and previous research using coarse-resolution data, particularly in alpine forests and marsh ecosystems, highlight the need for long-term high-resolution datasets to enhance ecological monitoring and conservation planning.
- Long-term remote sensing monitoring has emerged as a crucial tool for complementing the monitoring of protected areas and assessing the effectiveness of management actions.

## Supplementary Information

Below is the link to the electronic supplementary material.

Supplementary file1 (PDF 3592 KB)

## Data Availability

The data that support the findings of this study are available from the corresponding author upon reasonable request.
