# Supplementary material for: Long-term vegetation dynamics in Spain’s National Park Network: insights from remote sensing data
Source: Environ Monit Assess. 2025 Jun 19;197(7):767. doi: 10.1007/s10661-025-14233-w (PMC12176930; doi:10.1007/s10661-025-14233-w)
Supplement: Supplementary file 1 — Supplementary file1 (PDF 3592 KB) [file 10661_2025_14233_MOESM1_ESM.pdf]

Long-Term Vegetation Dynamics in Spain's National Park Network: A Remote Sensing Approach

Magí Franquesa[a,b], Maria Adell-Michavila[a,b], Sergio M. Vicente-Serrano[a,b]

[a]Instituto Pirenaico de Ecología, Consejo Superior de Investigaciones Científicas (IPE-CSIC), Zaragoza, 50059, Spain
[b]Laboratorio de Climatología y Servicios Climáticos (LCSC), CSIC-Universidad de Zaragoza, Spain

Corresponding author: Magí Franquesa (magi.franquesa@ipe.csic.es)

Supplementary material

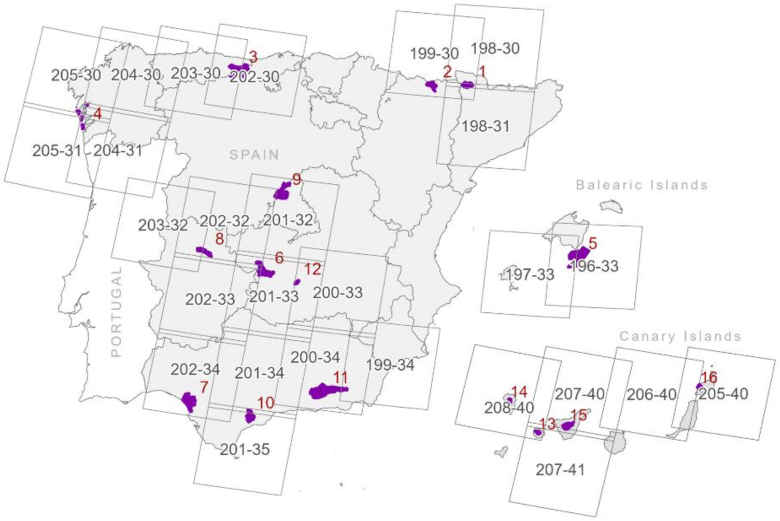

	National Park	Landsat Scene (Path-Row)	L5	L7	L8	Total
1	Aigüestortes	198-30, 198-31,199-30	1,014	1,003	577	2,594
2	Ordesa	199-30	681	696	376	1,753
3	Picos de Europa	202-30	592	571	374	1,537
4	Islas Atlánticas	204-30, 204-31, 205-30, 205-31	1,085	1,099	649	2,833
5	Cabrera	196-33, 197-33	751	661	396	1,808
6	Cabañeros	201-33, 202-32, 202-33	1,105	1,010	830	2,945
7	Doñana	202-34	753	753	428	1,939
8	Monfragüe	202-32	1066	953	620	2,639
9	Guadarrama	201-32	1229	1,196	817	3,242
10	Sierra de las Nieves	201-35	759	727	433	1,919
11	Sierra Nevada	200-34	798	781	654	2,233
12	Tablas de Daimiel	200-33, 201-33	732	715	407	1,854
13	Garajonay	207-40, 208-40	132	348	866	1,346
14	Taburiente	208-40	38	93	208	339
15	Teide	207-40	79	258	459	796
16	Timanfaya	205-40	123	530	430	1,083
	**Total**		**10,937**	**11,394**	**8,524**	**30,860**

Fig. S1 Landsat scenes (Path-Row) covering the 16 national parks. The table below provides the total count of valid images per park across all three Landsat satellites from 1984 to 2023, filtered to include only those with cloud cover below 80%. Total images used in the analysis are listed by satellite and by park

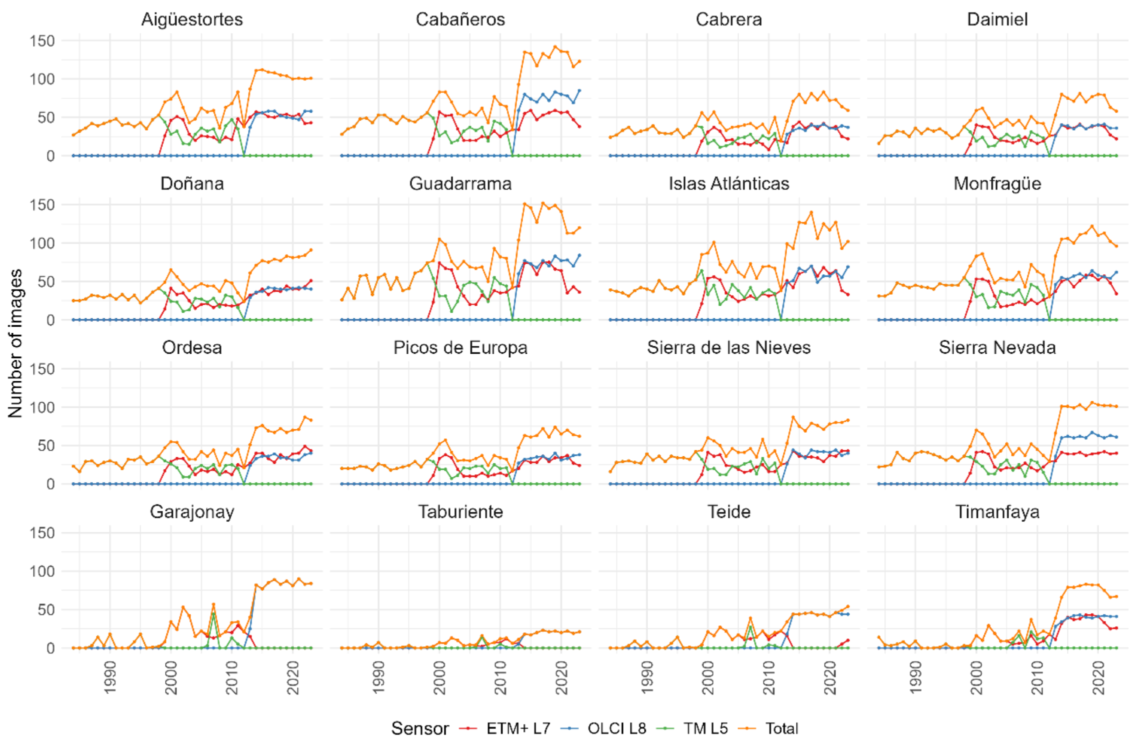

Fig. S2 Temporal distribution of available Landsat images (L5, L7, and L8) from 1984 to 2023 for each of the 16 national parks. The graph shows the number of valid images per year for each park, filtered by an 80% cloud cover threshold

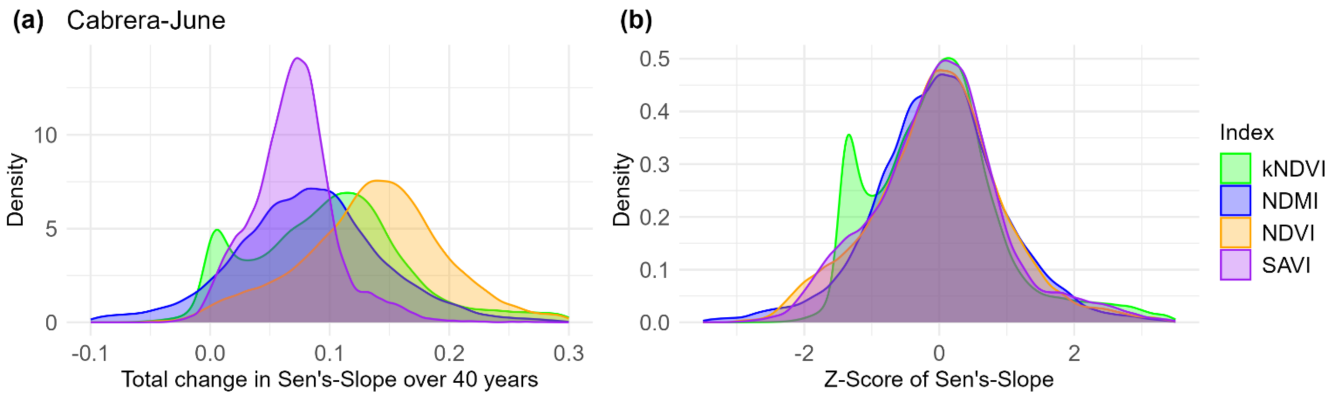

Fig. S3 Density plot of the total change in Sen's-Slope (magnitude) over 40 years for different vegetation indices in Cabrera National Park for June (a). Density plot of the corresponding Z-scores of these slopes (b). The first plot illustrates the actual magnitude of vegetation changes across NDVI, kNDVI, SAVI, and NDMI indices, while the second plot shows the standardized Z-scores, enabling a direct comparison by normalizing the slope values across all indices

Table S1 Reclassification of Natural Vegetation Systems in Spain's National Parks. This table presents the reclassification of Natural Vegetation Systems (SNV) from the official Cartographic Data Model of Natural Vegetation Systems used in Spain's National Parks Network. The table includes the original SNV identifier (Id_SNV), the reclassified category identifier (Id_Class), the name of the reclassified system (Class), and its description (Descripción/Description), which retains both the original Spanish classification and its English translation. This reclassification was performed to facilitate the analysis of vegetation trends by grouping ecologically similar systems

Id_SNV	Id_Class	Class	Descripción/Description
111100000	AlpConF	Alpine coniferous forest	Sistemas ligados a los bosques aciculifolios (coníferas) suboreales y/o boreoalpinos y sus variantes oromediterráneas<br>Systems associated with sub-boreal and/or boreo-alpine needleleaf (conifer) forests and their oro-Mediterranean variants
111200000	TempDecF	Temperate broadleaf deciduous forest	Sistemas ligados a los bosques planocaducifolios templados eurosiberianos y excepcionalmente mediterráneos<br>Systems associated with temperate Eurosiberian broadleaf deciduous forests and exceptionally Mediterranean
111300000	SMedMarF	sub-Mediterranean marcescent sub-sclerophyllous forests	Sistemas ligados a los bosques subesclerófilos marcescentes submediterráneos<br>Systems associated with sub-Mediterranean sub-sclerophyllous marcescent forests
111400000	MedSclF	Mediterranean sclerophyllous forest	Sistemas ligados a los bosques esclerófilos mediterráneos<br>Systems associated with Mediterranean sclerophyllous forests
111600000	MedConF	Mediterranean coniferous forests	Sistemas ligados a los bosques de cupresáceas o coníferas mediterráneas xerófilas<br>Systems associated with xerophytic Mediterranean Cupressaceae or conifer forests
111R00000	RipF	Riparian Forest	Sistemas ligados a bosque de ribera<br>Systems associated with Riparian Forest
112100000	AridScr	Arid Scrublands	Sistemas ligados a la vegetación ibérica esteparia-árida<br>Systems associated with Iberian steppe-arid vegetation
113100000	AlpScrGr	Alpine Scrublands and Grasslands	Matorrales subalpinos u oromediterráneos culminícolas<br>Subalpine and oro-Mediterranean summit shrublands
113200000			Pastizales circumárticos y eurosiberianos<br>Circum-Arctic and Eurosiberian grasslands
113300000			Pastizales psicroxerófilos oromediterráneos (dominio de Festuca spp.) y estepa leñosa de altura<br>Oro-Mediterranean psychroxerophilous grasslands (dominated by Festuca spp.) and high-altitude woody steppe
121000000	SaltMar	Salt marshes	Sistemas naturales ligados a hábitats costeros<br>Systems associated with coastal habitats
122000000	DuneSand	Dunes and sands	Sistemas naturales ligados a dunas marítimas y arenales continentales<br>Systems associated with maritime dunes and inland sandy areas
123000000	HaloVeg	Halophilous vegetation	Sistemas naturales ligados a vegetación halófila y gipsófila<br>Systems associated with halophytic and gypsophilous vegetation
124000000	HydroRipVeg	Hydrophilous and riparian vegetation	Vegetación hidrófila o higrófila y ribereña. Hábitats de aguas dulces<br>Hygrophilous, hydrophilic, and riparian vegetation (Freshwater habitats)
124500000	Peatland	Peatland	Turberas<br>Peatlands
125000000	RockScreeVeg	Rocky and Scree vegetation	Vegetación rupícola, saxícola o fisurícola, y de pedreras inestables<br>Rupicolous, saxicolous, fissuricolous, and unstable scree vegetation
125100000			Roquedos con vegetación casmofítica, fisurícola o espeluncícola<br>Rocky areas with chasmophytic, fissuricolous, or cave-dwelling vegetation

125200000			Vegetación ligada a gleras canchales y pedregales móviles<br>Vegetation associated with rockslides, scree slopes, and mobile stonefields
211000000	Shrub	Shrublands	Formaciones arbustivas<br>Shrubland Formations
211100000	Shrub	Shrublands	Espinares, rosaledas, con majuelos<br>Spiny shrublands, rose thickets, and hawthorn scrub
211300000	Shrub	Shrublands	Enebrales de Juniperus oxycedrus<br>Juniper woodlands (Juniperus oxycedrus stands)
212100000	MedScrub	Mediterranean scrublands	Matorrales ibéricos<br>Iberian shrublands
212110000	MedScrub	Mediterranean scrublands	Matorrales mediterráneos acidófilos<br>Acidophilous Mediterranean shrublands
212120000	MedScrub	Mediterranean scrublands	Matorrales mediterráneos basófilos<br>Basophilous Mediterranean shrublands
212130000	AtlScrub	Atlantic scrublands	Matorrales atlánticos<br>Atlantic shrublands
213100000	Grass	Mediterranean grasslands	Pastizales mediterráneos<br>Mediterranean grasslands
213200000	Grass	Atlantic grasslands	Pastizales atlánticos<br>Atlantic grasslands
213400000	Grass	Ruderal vegetation	Comunidades ruderales y arvenses ligadas a las actividades antropozoógenas<br>Ruderal and arable weed communities associated with anthropogenic and zoogenic activities
300000000	Dehesa	Dehesas (woody savanna)	Sistemas seminaturales<br>Semi-natural systems
900000000	ArtSurf	Artificial surfaces	Sistemas antrópicos<br>Anthropic systems
911000000	WaterSurf	Water surfaces	Sistemas abióticos<br>Artificial systems
912000000	BareArea	BareArea	Glaciares<br>Glaciers
913000000	BareArea	Bare areas	Ramblas y playas arenosas o pedregosas desiertas<br>Dry riverbeds and barren sandy or rocky beaches
914000000	BareArea	Bare areas	Roquedo sin vegetación<br>Bare rock without vegetation
922000000	Crop	Crops	Sistemas antrópicos<br>Anthropic Systems
921000000	Reforestation	Reforestation	Repoblaciones<br>Reforested areas
923000000	ArtSurf	Artificial surfaces	Sistemas artificiales<br>Artificial Systems
940000000	BareArea	Bare areas	Fangos mareales<br>Tidal mudflats

28

Table S2 Seasonal variability (Δavg) of vegetation indices across Spanish National Parks. The table presents the range of monthly mean values (Δavg), calculated as the difference between the highest and lowest average monthly values, for NDVI, kNDVI, SAVI, and NDMI across the 12 Spanish National Parks. Higher values indicate greater seasonal fluctuations in vegetation activity, while lower values reflect more stable conditions throughout the year

National Park	NDVI	KNDVI	SAVI	NDMI
Aigüestortes	0.42	0.29	0.28	0.70
Cabañeros	0.12	0.09	0.04	0.11
Cabrera	0.12	0.07	0.05	0.15
Islas Atlánticas	0.16	0.04	0.03	0.12
Las Tablas de Daimiel	0.10	0.23	0.11	0.15
Doñana	0.09	0.08	0.04	0.28
Sierra de Guadarrama	0.20	0.18	0.14	0.30
Monfragüe	0.16	0.08	0.05	0.14
Ordesa y Monte Perdido	0.42	0.32	0.31	0.53
Picos de Europa	0.41	0.52	0.33	0.28
Sierra Nevada	0.18	0.08	0.13	0.36
Sierra de las Nieves	0.15	0.09	0.05	0.11

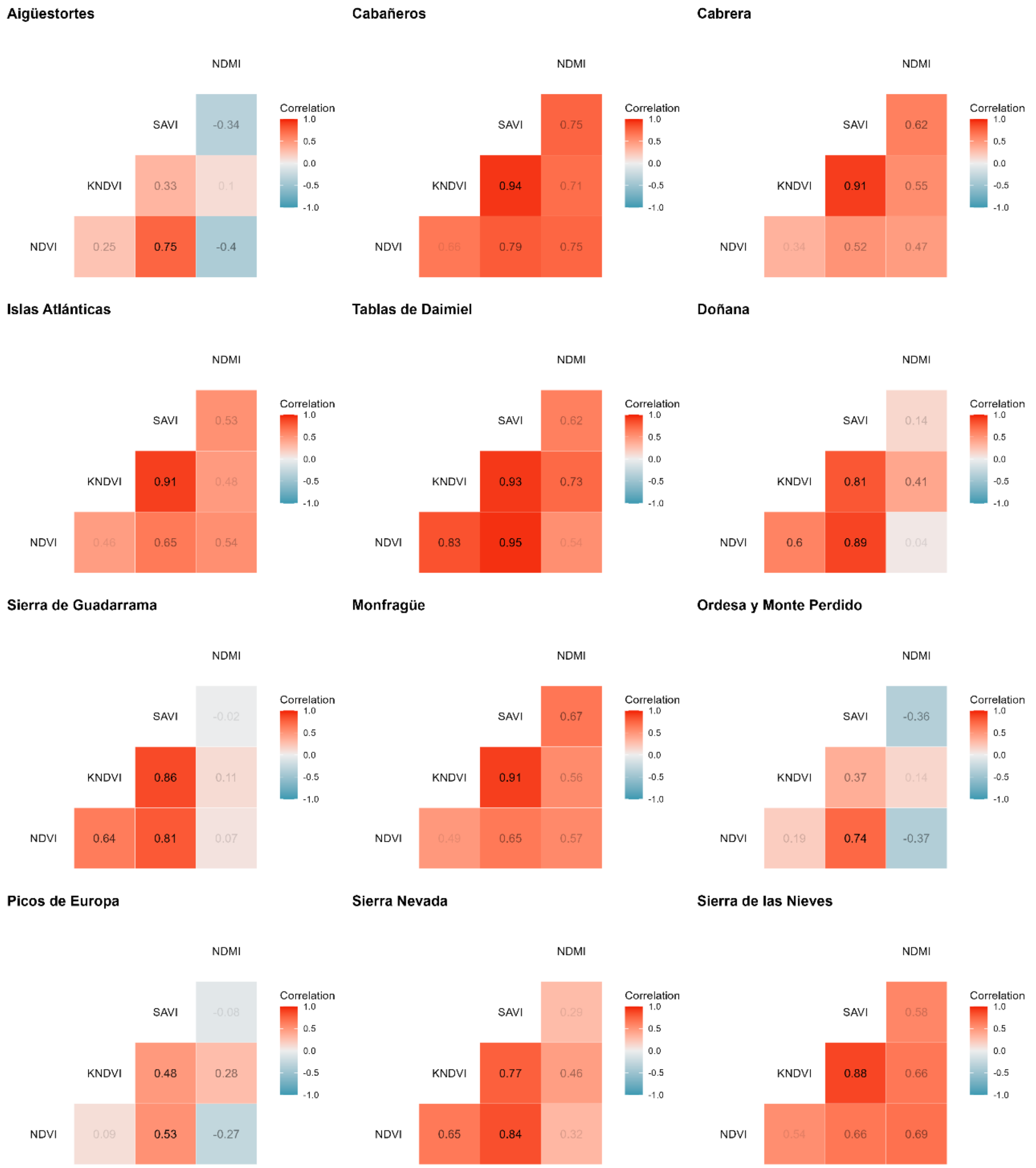

Fig. S4 Correlation between the slope of vegetation indices trends (NDVI, KNDVI, SAVI, and NDMI) for the month of January across 12 Spanish national parks. Each subplot represents a park, and the values indicate the Pearson correlation coefficients between the slopes of the indices. Strong correlations (close to ±1) suggest similar trends in vegetation dynamics captured by the indices

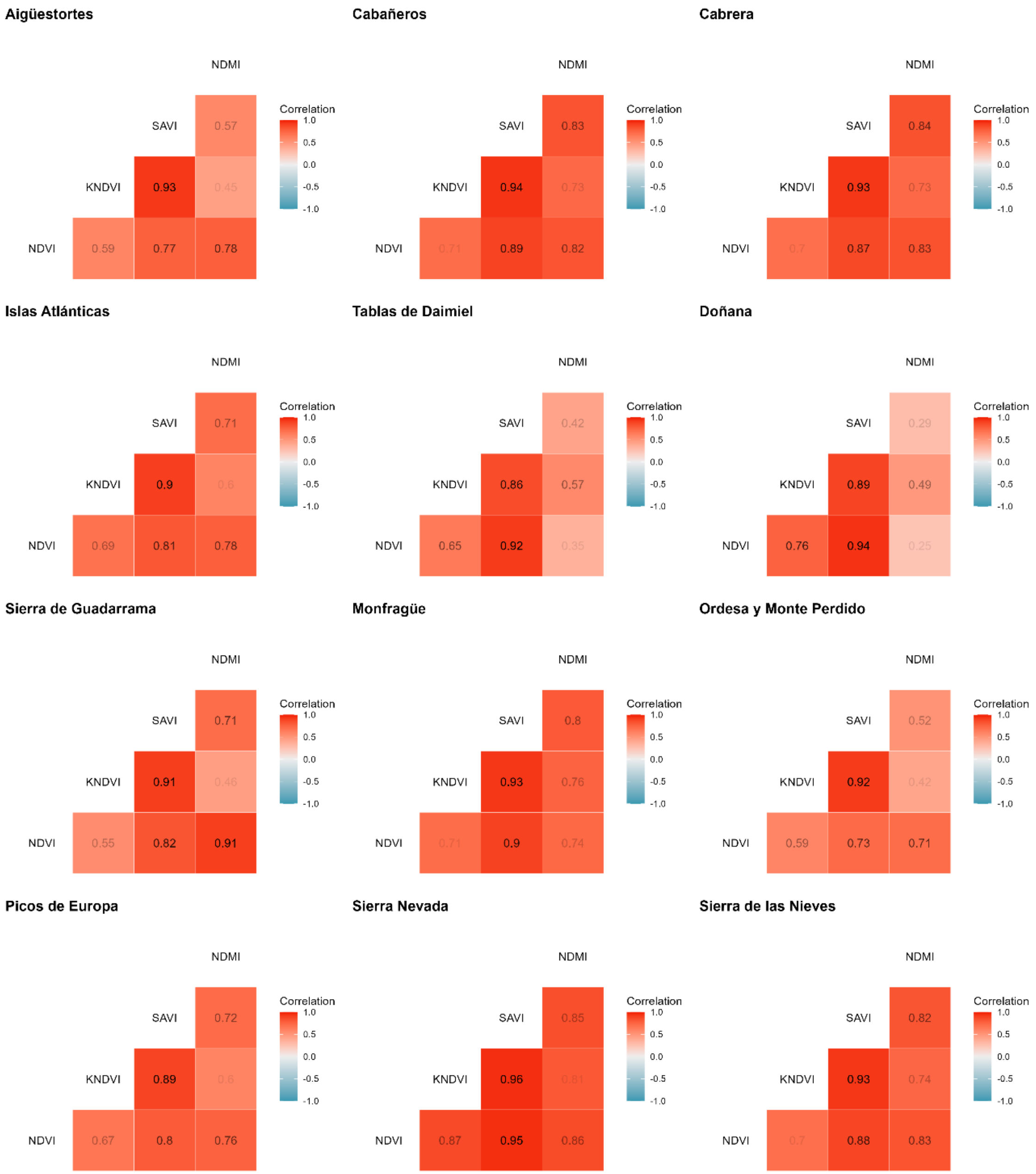

Fig. S5 Correlation between the slope of vegetation indices trends (NDVI, KNDVI, SAVI, and NDMI) for the month of July across 12 Spanish national parks. Each subplot represents a park, and the values indicate the Pearson correlation coefficients between the slopes of the indices. Strong correlations (close to ±1) suggest similar trends in vegetation dynamics captured by the indices

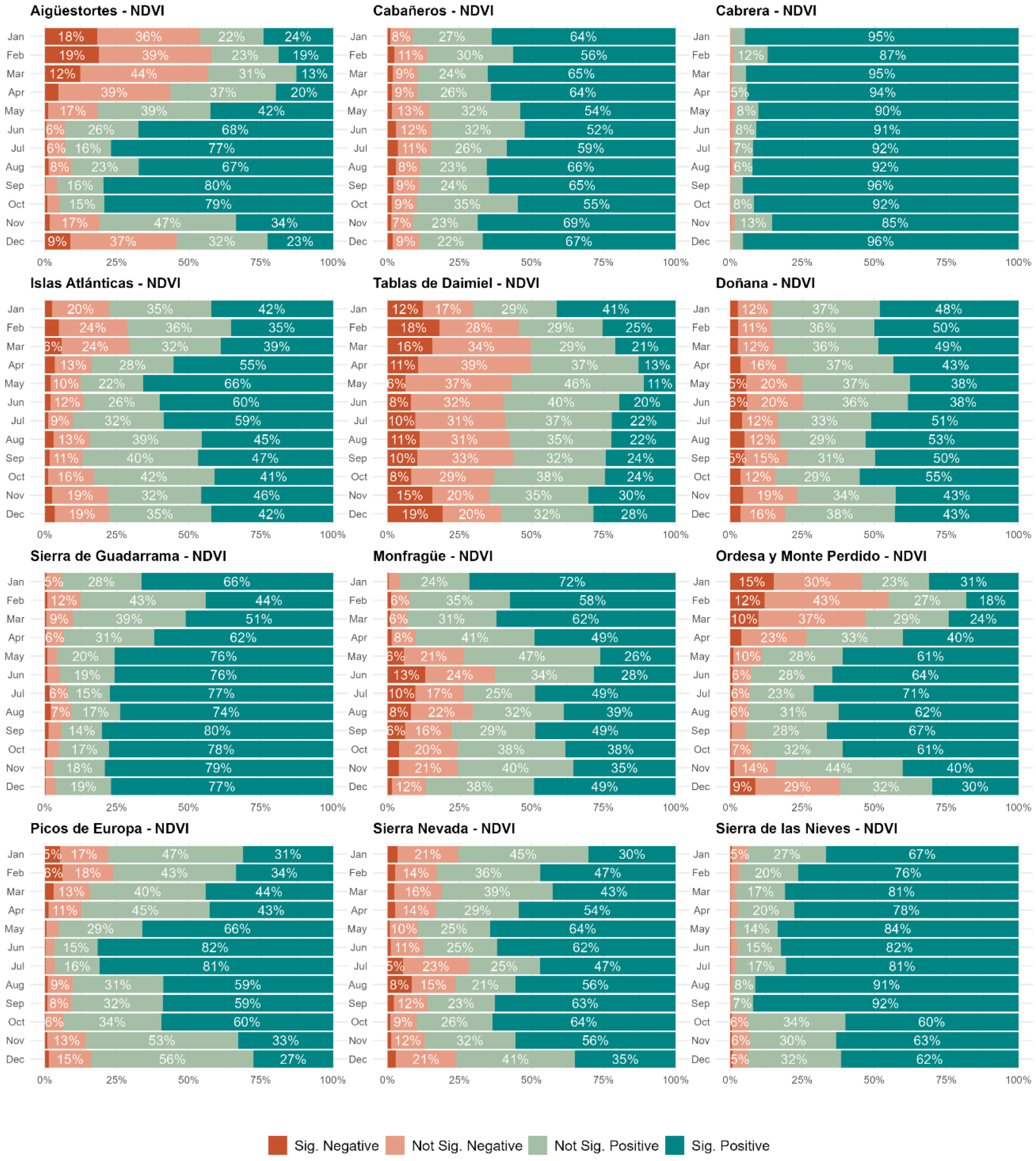

Fig. S6 Monthly percentage distribution of significant and non-significant NDVI trends across 12 Spanish national parks. Each horizontal bar represents the proportion of pixels with significant positive, non-significant positive, non-significant negative, and significant negative trends for each month

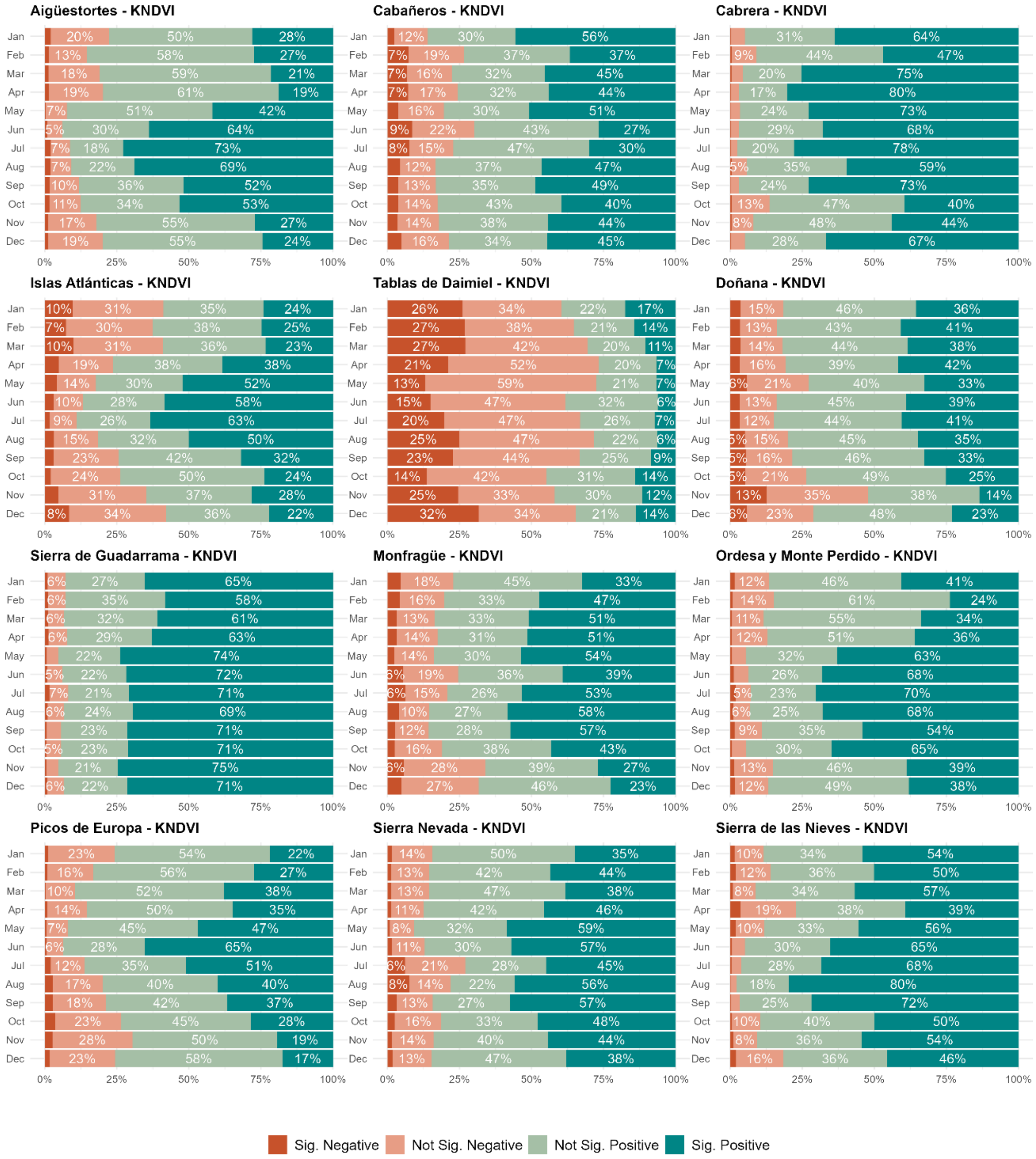

Fig. S7 Monthly percentage distribution of significant and non-significant kNDVI trends across 12 Spanish national parks. Each horizontal bar represents the proportion of pixels with significant positive, non-significant positive, non-significant negative, and significant negative trends for each month

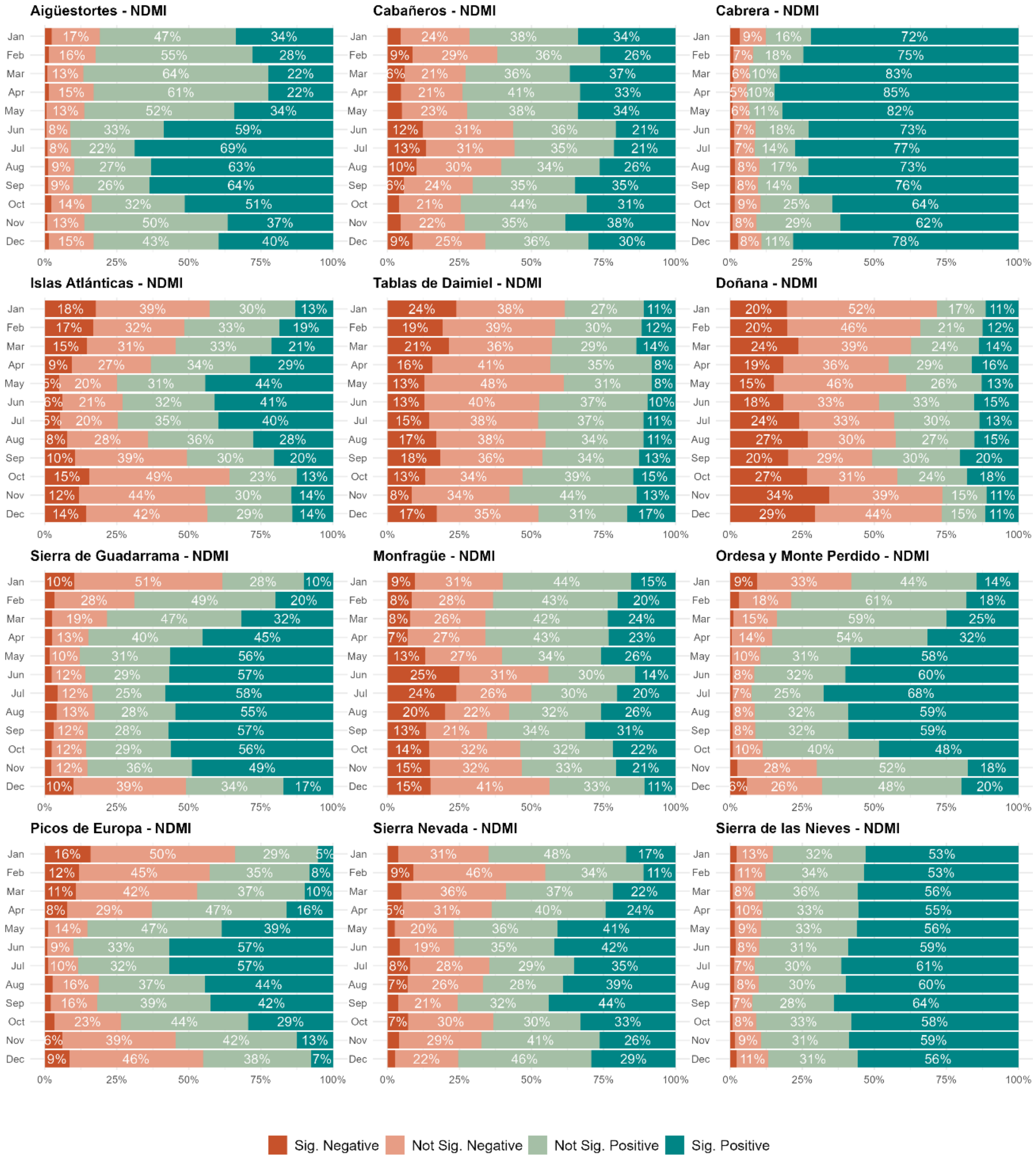

Fig. S8 Monthly percentage distribution of significant and non-significant NDMI trends across 12 Spanish national parks. Each horizontal bar represents the proportion of pixels with significant positive, non-significant positive, non-significant negative, and significant negative trends for each month

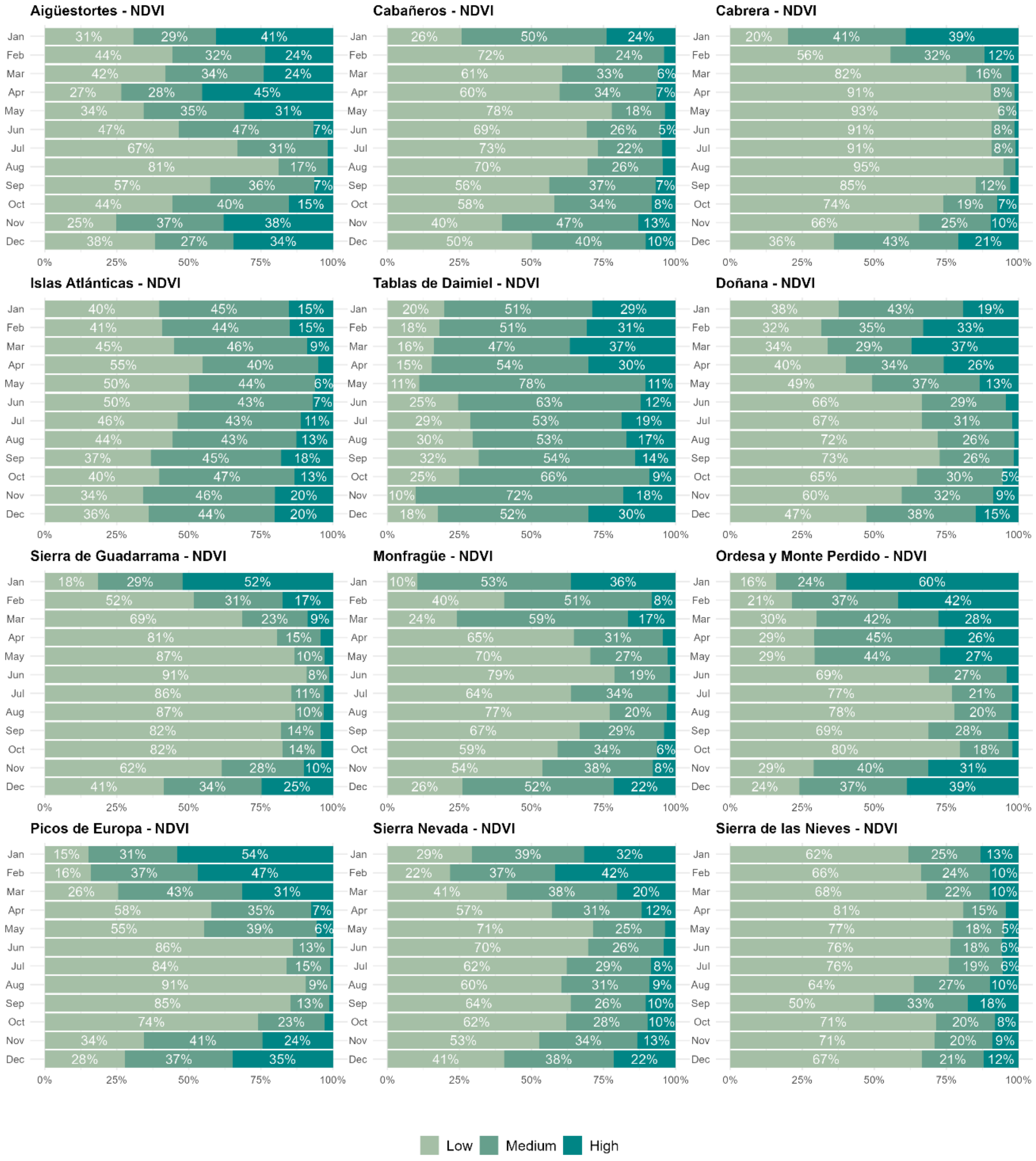

Fig. S9 Distribution of significant positive vegetation trends classified by magnitude of change (NDVI) across 12 Spanish national parks on a monthly basis. The magnitude of change is expressed as the total slope change over 40 years, classified into three categories: Low (Z-score < 0.5), Medium (0.5 ≤ Z-score < 1.5), and High (Z-score ≥ 1.5). These three categories are based on z-scores calculated independently for each park

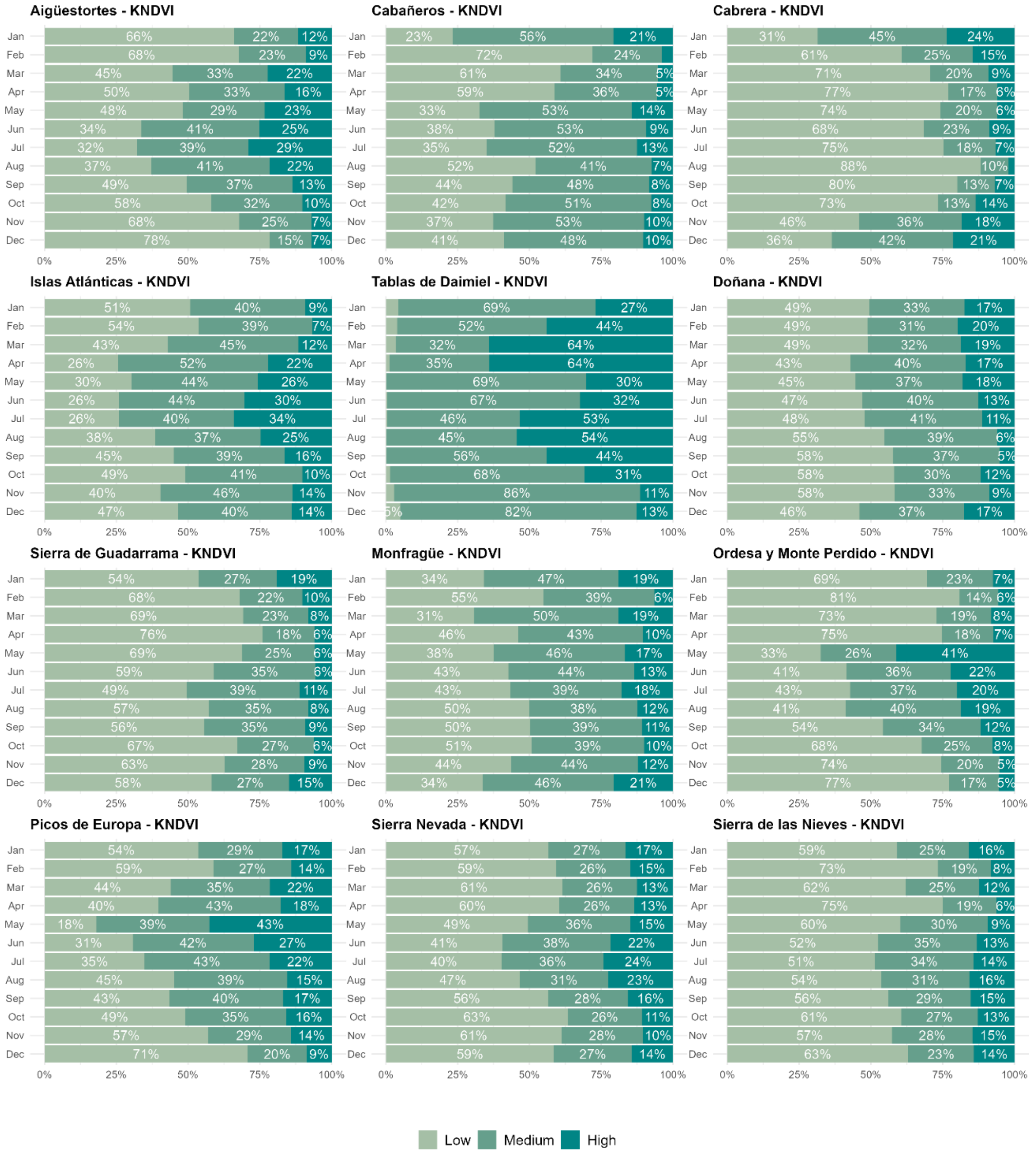

Fig. S10 Distribution of significant positive vegetation trends classified by magnitude of change (kNDVI) across 12 Spanish national parks on a monthly basis. The magnitude of change is expressed as the total slope change over 40 years, classified into three categories: Low (Z-score < 0.5), Medium (0.5 ≤ Z-score < 1.5), and High (Z-score ≥ 1.5). These three categories are based on z-scores calculated independently for each park

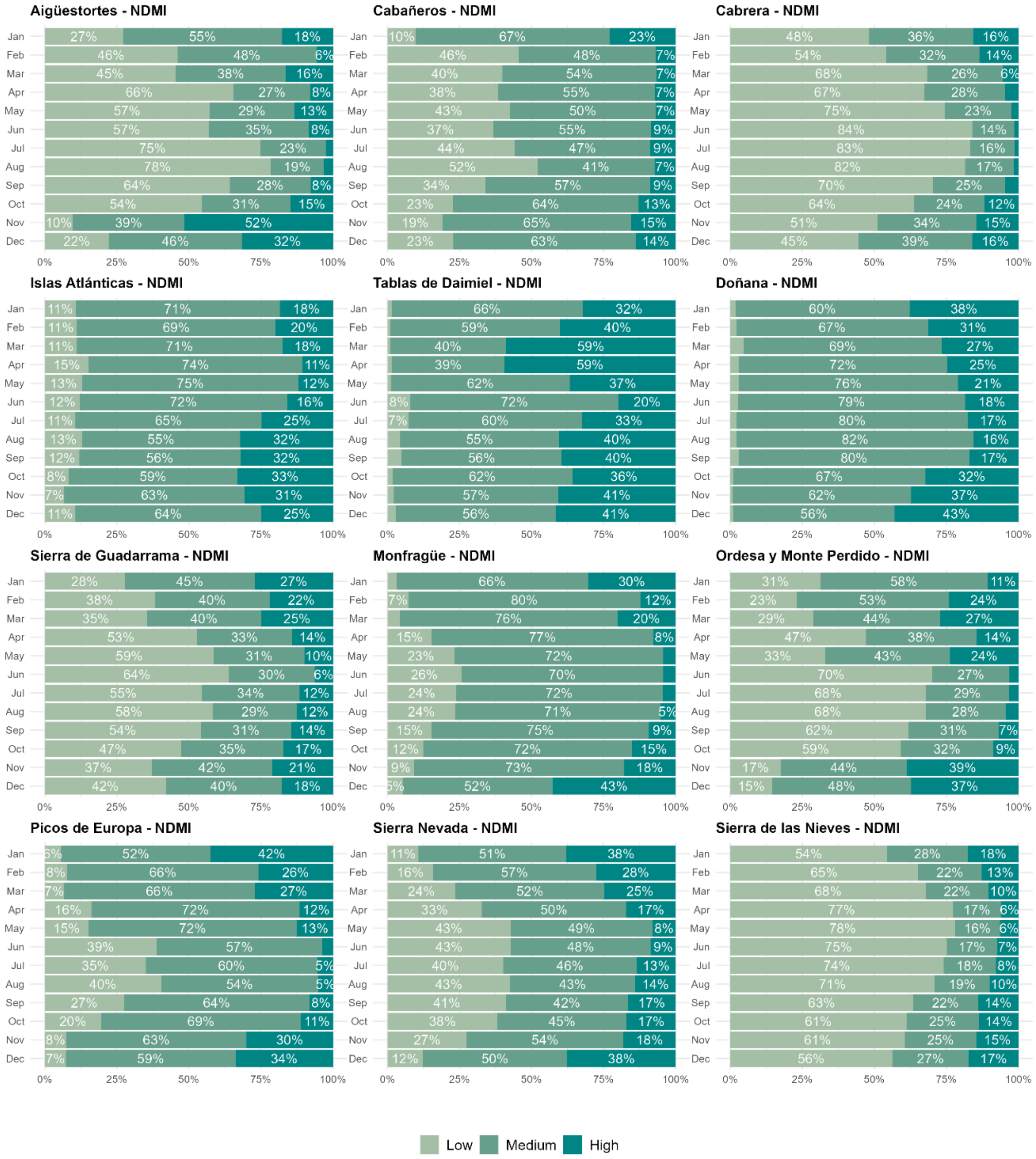

Fig. S11 Distribution of significant positive vegetation trends classified by magnitude of change (NDMI) across 12 Spanish national parks on a monthly basis. The magnitude of change is expressed as the total slope change over 40 years, classified into three categories: Low (Z-score < 0.5), Medium (0.5 ≤ Z-score < 1.5), and High (Z-score ≥ 1.5). These three categories are based on z-scores calculated independently for each park.

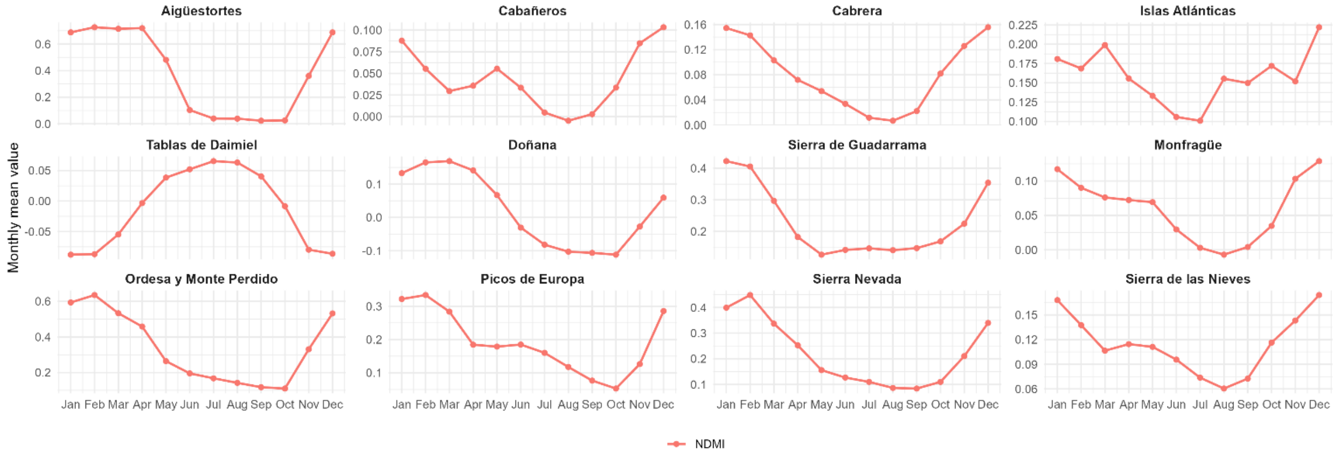

Fig. S12 Monthly mean values of the NDMI index for 12 Spanish national parks over a period of 40 years. The x-axis represents months (January to December), and the y-axis indicates the mean NDMI values. Each panel corresponds to a specific national park, with variable y-axis scales to better highlight seasonal vegetation dynamics

Supplementary Method – Validation of the Gap-Filling Methodology

To evaluate the performance and consistency of the NDVI gap-filling procedure, we implemented a validation strategy at the pixel level focused primarily on the temporal integrity of the reconstructed series. Rather than prioritizing the recovery of individual missing values, our objective was to ensure that the reconstructed time series retained the original signal's structural characteristics and long-term dynamics.

For each national park, we randomly selected 100 locations with at least 200 valid observations. To test the method's accuracy in recovering missing values, we applied a cross-validation strategy in which 20% of the valid NDVI values were randomly masked, and the reconstruction function was applied. The reconstructed values at the masked positions were then compared to the original values using standard metrics such as RMSE, R^2, bias, and Willmott's d.

More importantly, we evaluated the coherence between the complete reconstructed series and the original series (excluding only native gaps). This evaluation captured how well the gap-filling procedure preserved the shape, variability, and trends of the original signal. We computed the same metrics over the entire overlapping portions of the series and estimated the absolute difference in slope between the original and reconstructed time series using the Theil–Sen estimator. This slope difference was used to assess whether long-term temporal trends were maintained.

The results confirm that the reconstruction method provides robust estimates at the series level. Despite some expected discrepancies at the level of individual reconstructed values, the full time series remain highly consistent with the original data, with low RMSE and bias, strong agreement ($d > 0.92$), and negligible distortion in trend magnitude (Table S3, Fig. S13). This suggests that the reconstruction approach is well suited for applications where temporal coherence and trend detection are critical, as in our long-term vegetation indices analyses.

Table S3. Summary of validation metrics for the NDVI reconstruction across the 12 Spanish national parks. The left block shows pixel-wise assessment metrics based on the comparison between original and reconstructed NDVI values at masked positions (20% randomly removed). The right block summarizes the assessment over the entire time series (excluding native gaps), highlighting the preservation of temporal structure and trends. RMSE: root mean square error; R^2: coefficient of determination; Bias: mean error; Willmott d: index of agreement; SlopeDiff: absolute difference in Theil–Sen slope estimates.

	Pixel-wise assessment				Time series (ts) assessment			
**National Park**	**RMSE**	**R^2**	**Bias**	**Willmot_d**	**RMSE(ts)**	**R^2(ts)**	**Willmot_d(ts)**	**SlopeDiff**
Aigüestortes	0.141	0.663	0.010	0.889	0.063	0.925	0.980	0.000026
Cabañeros	0.092	0.339	0.011	0.715	0.041	0.841	0.956	0.000013
Cabrera	0.101	0.296	0.008	0.708	0.045	0.834	0.955	0.000018
Islas Atlánticas	0.233	0.076	0.011	0.473	0.104	0.732	0.923	0.000043
Las Tablas de Daimiel	0.097	0.605	0.012	0.871	0.043	0.915	0.978	0.000015
Doñana	0.101	0.476	0.011	0.798	0.045	0.881	0.967	0.000014
Sierra de Guadarrama	0.120	0.257	0.007	0.676	0.054	0.824	0.952	0.000025
Monfragüe	0.093	0.383	0.011	0.758	0.041	0.856	0.961	0.000015
Ordesa y Monte Perdido	0.161	0.443	0.013	0.778	0.072	0.868	0.964	0.000034
Picos de Europa	0.175	0.473	0.012	0.800	0.078	0.882	0.968	0.000026
Sierra Nevada	0.111	0.343	0.011	0.724	0.050	0.845	0.957	0.000018
Sierra de las Nieves	0.081	0.448	0.011	0.784	0.036	0.876	0.966	0.000020

The results reveal a clear distinction between the performance of the gap-filling procedure when evaluated at the individual value level (Pixel-wise assessment) and across the full time series (Time series assessment). Pixel-wise metrics (RMSE, R^2, Bias, and Willmott's d) show moderate agreement between observed and reconstructed NDVI values, with RMSEs typically below 0.15 and biases close to zero. However, R^2 values are generally low (especially in parks like Islas Atlánticas, Cabrera or Guadarrama), reflecting the inherent difficulty of predicting isolated values in highly variable vegetation signals.

Time series-based metrics provide a much stronger validation of the reconstruction approach. RMSE(ts) values are consistently low (typically < 0.08), R^2(ts) values range from 0.732 (Islas Atlánticas) to 0.925 (Aigüestortes), indicating substantial agreement between original and reconstructed series. Willmott’s d(ts), which measures the similarity between both original and reconstructed series, remains high (all > 0.92), confirming excellent agreement. Crucially, the SlopeDiff metric is near zero (typically < 0.00003), suggesting that long-term trends in vegetation dynamics are faithfully preserved during reconstruction. Together, these metrics support the reliability of the gap-filling method for long-term vegetation indices analysis, especially when trend preservation and structural coherence are more critical than pointwise reconstruction accuracy.

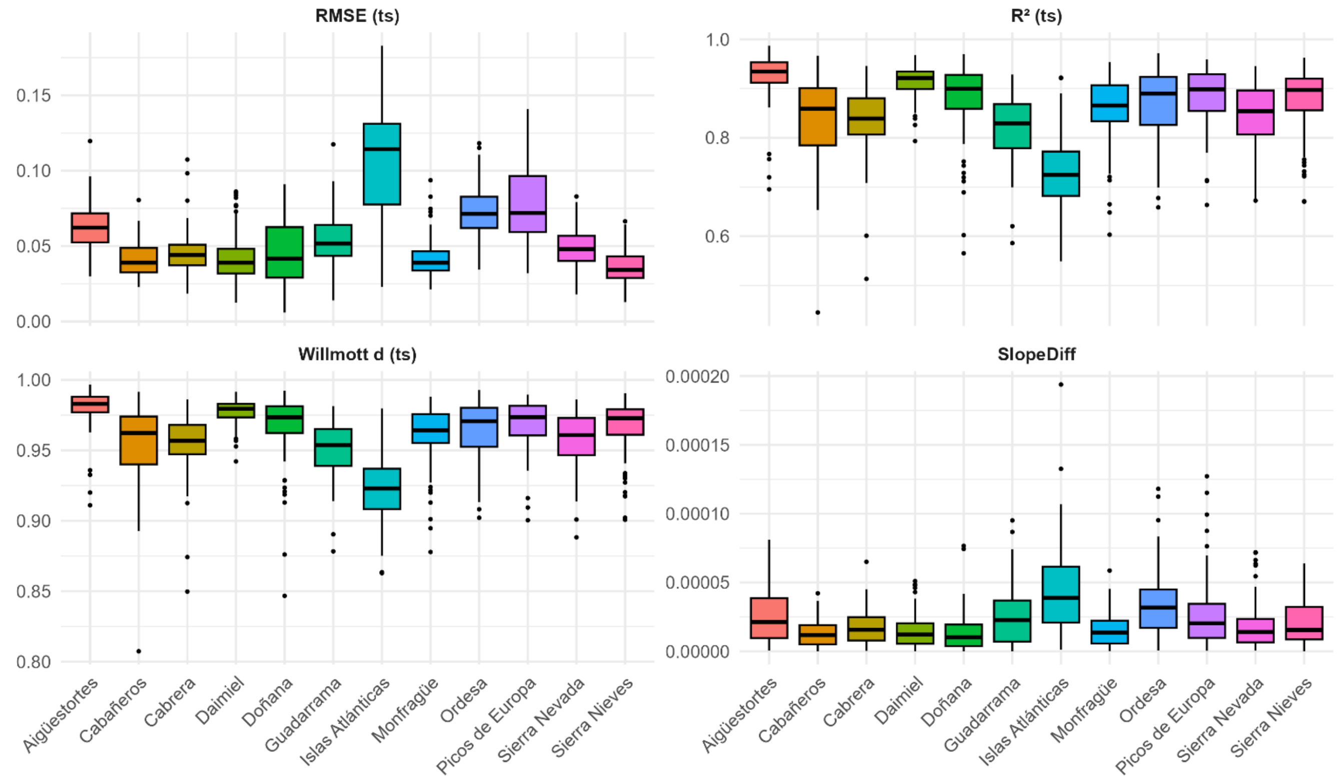

Fig. S13. Distribution of validation metrics for the full reconstructed NDVI time series across 100 sampled pixels per national park. The metrics include root mean square error (RMSE), coefficient of determination (R^2), Willmott's agreement index (d), and absolute difference in trend slope (SlopeDiff) between the original and reconstructed time series. Each boxplot represents the variability of the corresponding metric across individual pixels within each park.
